# Pan-cancer analysis of trophinin-associated protein with potential implications in clinical significance, prognosis, and tumor microenvironment in human cancers

**DOI:** 10.3389/fonc.2022.971618

**Published:** 2022-11-07

**Authors:** Zhenfen Li, Zhangya Pu, Ziyue Yang, Yuanyuan Zhu, Ying Deng, Ning Li, Fang Peng

**Affiliations:** ^1^ Department of Blood Transfusion, Clinical Transfusion Research Center, Xiangya Hospital, Central South University, Changsha, Hunan, China; ^2^ National Health Commission (NHC) Key Laboratory of Cancer Proteomics, Xiangya Hospital, Central South University, Changsha, Hunan, China; ^3^ National Clinical Research Center for Geriatric Disorders, Xiangya Hospital, Central South University, Changsha, China; ^4^ Department of Infectious Diseases and Hunan Key Laboratory of Viral Hepatitis, Xiangya Hospital, Central South University, Changsha, China; ^5^ Department of Scientific Research Management, Ningxiang People’s Hospital, Hunan University Traditional Chinese Medicine, Ningxiang, Changsha, Hunan, China

**Keywords:** trophinin-associated protein, pan-cancer, tumor microenvironment, biomarker, immunological, prognostic

## Abstract

**Background:**

Trophinin-associated protein (TROAP), a cytoplasmic protein, is essential for microtubule cytoskeleton assembly. Mounting evidence demonstrates the vital role of TROAP in regulating the proliferation and migration of cells, but it is unclear how it contributes to cancer progression.

**Methods:**

The online portals of GEPIA2, Cancer Cell Line Encyclopedia, UALCAN, Human Protein Atlas, and PrognoScan were used to analyze TROAP expression in various tumors and further evaluate its correlation with prognosis. With Western blot and quantitative real-time PCR analysis, we validated TROAP expression levels in hepatocellular carcinoma (HCC) and colorectal cancer (CRC). Ten pairs of HCC and CRC tissues were selected for immunohistochemistry to determine TROAP expression levels in tumors and adjacent tissues, respectively. TROAP knockdown in CRC and HCC cells to verify its role in malignant phenotypes. The genomic and post-transcriptional alterations of TROAP in tumors were determined using the cBioPortal and SangerBox databases. Also, TISIDB was used to investigate the relationship between TROAP expression and tumor microenvironment(TME) among different cancer types. Moreover, a correlation was found between the expression of TROAP and drug sensitivity using GSCALite and CellMiner databases.

**Results:**

TROAP expression was significantly upregulated in most cancer types, which is consistent with our validated experimental results in HCC and CRC cells, and immunohistochemistry results. And a poor prognosis was linked to TROAP aberrant expression. Our findings indicated that malignant phenotypes and tumorigenesis induced by TROAP could be due to an activation of the PI3K/Akt/GSK-3β signaling pathway. Furthermore, we found a correlation between TROAP expression and genomic and post-transcriptional alterations in various tumors, including tumor mutation burden, and microsatellite instability. Next, we demonstrated that TROAP expression was associated with the infiltration of immune cells, such as neutrophils and macrophages, and correlated with immunomodulation-related genes in the TME. Additionally, the potential role of TROAP expression in predicting the sensitivity of drugs, including melphalan and chlorambucil, was demonstrated.

**Conclusions:**

Collectively, these findings indicated a significant correlation between TROAP expression and malignant phenotype, functional mechanism, survival possibility, TME, therapeutic potential, and prediction of drug sensitivity in various cancers. Hence, TROAP is a promising biomarker and therapeutic target for predicting cancer outcomes.

## Introduction

According to current estimates of global mortality data, cancer is the second leading cause of death globally and will likely become the first in 2060 (1). However, this method lacks a uniform and effective solution (2). Immunotherapy has emerged as an effective new therapeutic strategy for antineoplastic treatment (3). In contrast to conventional therapies that show a broadly suppressive effect, immunotherapies that depend on the dynamic interaction of tumor and immune cells in the tumor microenvironment (TME) are more effective targeted therapies, in which therapeutic efficacy and reagent-related toxicities are more straightforward to monitor. Hence, further exploration of biomarkers correlated with the prognostic value and immunotherapeutic response for various cancers is warranted to maximize the clinical benefit for patients with cancer (4).

The TME is comprised of various cell types and extracellular components that surround tumor cells and are supported by a vascular network (5). The complexity of the TME is related to tumor growth, metastasis, and response to therapy. Many “hallmarks of cancer” are linked to the TME, including encouraging immunological escape and activating immune cells to support invasion and metastasis. Microsatellite instability (MSI) refers to cancers containing a defective mismatch repair mechanism leading to hypermutation and accumulation of mutations in monomorphic microsatellites, which are particularly prone to mismatch errors (6). MSI and tumor mutation burden (TMB) are indirect measures of tumor antigenicity generated by somatic tumor mutations, which are related to cancer development (7). A neoantigen (NEO) is a nascent antigen encoded by a mutated gene in tumor cells (8). These are new biomarkers for evaluating the therapeutic efficacy of immune checkpoint inhibitors (ICIs), which augment adaptive immunity (9). Dynamic changes in the TME lead to the selection of mutations in tumor cells, contributing to genomic instability. Therefore, a thorough investigation of the connection between the immune microenvironment and tumor genetic alterations as well as the identification of therapeutic targets in the TME may yield new molecular targets for anti-tumor immunotherapy (10).

In 1995, two human epithelial cell lines were used to clone the trophinin-associated protein (TROAP), formally named tastin by Fukuda et al (11, 12). Previous studies indicated that TROAP can act as a cell adhesion molecule and associate with trophinin and bystin to mediate initial attachment (13, 14). Moreover, TROAP levels quickly dropped following cell division and peaked in the G2/M phase. The absence of TROAP expression prevents mitotic blocking and induces the creation of multipolar spindles, whereas TROAP overexpression causes the production of monopolar spindles, showing that TROAP is involved in cell proliferation (15). Furthermore, TROAP participates in the invasion and migration of multiple cancers, for instance, lung, liver, and breast cancer (16–18). For example, in glioma, TROAP promotes malignant biological behavior and G1/S cell cycle arrest by activating the Wnt/β-catenin pathway (19). On the contrary, the biological role of TROAP in the progression of cancers and its correlation with prognosis, TME, and prediction of drug sensitivity is still unclear in the systematic analyses. Therefore, we evaluated cancer samples and adjacent normal tissues for TROAP expression differences and their correlation with prognosis in pan-cancer according to bioinformatic analyses. Additionally, we examined possible associations between TROAP expression and tumor-infiltrating lymphocytes (TILs) and immune subtypes in the TME. A further investigation was conducted to determine whether TROAP expression is associated with drug sensitivity.

## Methods

### Gene expression profiling interactive analysis (GEPIA2) and PrognoScan database

GEPIA2 (http://gepia2.cancer-pku.cn/#analysis) is a server that allows users to analyze gene expression data from the Cancer Genome Atlas (TCGA) (https://cancergenome.nih.gov/) and the genotype-tissue expression (GTEx) database (https://www.gtexportal.org) for tumors and normal tissues (20). The difference in TROAP transcriptional levels in normal and tumor samples and the correlation of its expression level and survival possibility in pan-cancer were determined using GEPIA2. In addition, gene expression and prognosis were assessed using the PrognoScan database (http://kmplot.com/analysis/), and TROAP’s prognostic value was validated in different cancers (21).

### BioGPS and cancer cell line encyclopedia database

BioGPS (http://biogps.org) was used to show the abundance of TROAP expression in different cells or tissues (22). The transcriptional data of TROAP in tumor cell lines were downloaded from the Cancer Cell Line Encyclopedia (CCLE) database (https://portals.broadinstitute.org/ccle) (23). Gene expression analyses were performed with the use of R software (version 4.0.3), and box plots were generated using the R Documentation package ggplot2 (v3.3.3).

### UALCAN portal

The UALCAN portal (http://ualcan.path.uab.edu/cgi-bin/ualcan-res.pl) was used to examine the gene expression and survival data of a variety of cancers from the TCGA database to estimate the effect of TROAP transcriptional expression on clinicopathological features (24).

### The human protein atlas (HPA)

The HPA database (http://www.proteinatlas.org/) contains numerous protein expression data based on multiple cancers’ immunohistochemistry (IHC) staining (25, 26). Hence, to investigate the protein expression of TROAP within tumor tissues and adjacent tissues, we retrieved immunohistochemical staining data from the HPA database.

### SangerBox portal

The SangerBox website (http://vip.sangerbox.com/home.html) was used to extract RNA sequencing data of pan-cancer and normal tissue samples from TCGA and GTEx databases for bioinformatics analyses, to investigate the association between TROAP expression and immune checkpoint inhibitors, survival, tumor mutation burden, microsatellite instability, neoantigen, mutant-allele tumor heterogeneity, methylation modifications, and tumor-infiltrating lymphocytes.

### cBioPortal

In cBioPortal (http://cbioportal.org) cancer genomic data based on TCGA cancer types can be explored and analyzed for free through its open-access website (27, 28). We used it to analyze the TROAP genomic alterations in pan-cancer.

### GSCALite

Gene Set Cancer Analysis (GSCALite) (http://bioinfo.life.hust.edu.cn/GSCA/#/) includes a mass of multi-dimensional genomic data from TCGA and abundant small-molecule drugs from the genomics of drug sensitivity in cancer (GDSC) and the cancer therapeutics response portal (CTRP) (29). It was used to examine the correlation between drug sensitivity and copy number variations (CNVs) of TROAP in different cancers.

### TISIDB

Integrated repository portal for tumor-immune system interactions (TISIDB) (http://cis.hku.hk/TISIDB/index.php) is an integrator for analyzing specific genes in tumor-immune interactions (30). It was used to explore the relationship between TROAP expression and molecular or immune subtypes of different cancers and conduct immune-related genes co-expressed with TROAP in 33 cancer types using Pearson’s correlation coefficient.

### CellMiner

CellMiner (https://discover.nci.nih.gov/cellminer/home.do) contains NCI-60 compound activity and the RNA sequencing expression profiles in various cancer cell lines (31, 32). We employed R packages including “impute,” “limma,” “ggplot2,” and “ggpubr” to determine the impact of TROAP expression on drug sensitivity.

### Tumor immune single-cell hub (TISCH)

TISCH is a scRNA-seq database focused on the tumor microenvironment (TME) (33). The integrated data resources will help to explore gene regulation and immune signals in TME and provide directions for the discovery of new drug targets.

### Cell culture, lentivirus production and transduction

The human colorectal cancer (CRC) (SW480 and SW620), normal colorectal mucosa (NCM460), hepatocellular carcinoma (HCC) (HepG2 and HCC-LM3 cells), and normal liver (LO2) cell lines were provided by the NHC Key Laboratory of Cancer Proteomics, Xiangya Hospital (Changsha, China). The cells were routinely maintained in Dulbecco’s minimal essential medium (DMEM, Gibco, USA) supplemented with 10% fetal bovine serum (FBS, Gibco, USA) and 1% penicillin-streptomycin (Gibco, USA) in a humidified incubator at 37°C and 5% CO2. Two short-hairpin RNAs (shRNAs) targeting TROAP were cloned into lentiviral interference vector pLKO.1-puro with the following primers: shTROAP-#1: 5′-CCTCCAACTCTGACCTCATAT-3′; shTROAP-#2: 5′-GCCCTGTGTTTCATTCCAGTT-3′. To produce lentiviral particles, 293T cells were co-transfected with pMD2.G (Addgene, #12259), psPAX2 (Addgene, #12260), and pLKO.1-puro-shControl or pLKO.1-puro-shTROAP lentiviral plasmids using Polyethylenimine Linear (PEI) in accordance with the manufacturer’s instructions. Viral particles were collected 48 and 72 hours after transfection. Viral supernatants were applied to HCC-LM3 and SW620 cells in the presence of 5 µg/mL polybrene, and transduced cells were selected with 2 μg/mL and 5 μg/mL puromycin, respectively.

### RNA extraction and quantitative real-time PCR (qRT-PCR)

TRIzol reagent (Invitrogen, USA) was used to extract total RNA from the cell lines. The quality was assessed using a NanoDrop™ 1000 (Thermo Fisher Scientific, USA). Complementary DNA was synthesized using a TaqMan MicroRNA Reverse Transcription Kit (Applied Biosystems, USA). qRT-PCR was performed using the miScript SYBR Green PCR kit (Qiagen) on an ABI7500 instrument (Applied Biosystems). GAPDH was used as the endogenous control for RNA quantification. The data were analyzed using the 2^–ΔΔCt^ method. The primer sequences were as follows: (TROAP) 5′-GTTTAACCGCCATCCACTGC-3′ and 5′-TCGAGTAATGTAGCCACAGGG-3′, (GAPDH) 5′-GAAACTGTGGCGTGATGGC-3′ and 5′-CCGTTCAGCTCAGGGATGAC-3′.

### Western blotting

Cells were collected and lysed with a buffer (RIPA, Beyotime, China) containing phenylmethanesulfonyl fluoride (PMSF, Sigma, USA) and protease inhibitor cocktail (APExBIO; K1007; USA). For western blotting, proteins from each sample were separated using 10% SDS-PAGE and then transferred onto a polyvinylidene fluoride (PVDF) membrane. The membrane was blocked with TBST containing 5% non-fat milk, and the membranes were incubated with primary antibodies overnight at 4°C. Subsequently, the membranes were incubated for 2 h with the corresponding secondary antibodies, including anti-rabbit and anti-mouse horseradish peroxidase (HRP)-linked IgG (Immunoway, 1:5000). The working dilutions of primary antibodies were: TROAP: 1:400 (Proteintech, 13634-1-AP) and GAPDH:1:5000 (Abmart, M20006). Phospho-Akt (Ser473): 1:1000(Cell Signaling Technology, #4060); Akt (pan) (C67E7):1:1000(Cell Signaling Technology, #4691); Phospho-GSK-3β (Ser9):1:1000(Cell Signaling Technology, #5558); GSK-3β:1:1000(Proteintech, 22104-1-AP); and PI3K p110(beta) (Proteintech, 20584-1-AP).

### Cell proliferation, wound healing assay and transwell assay

Assaying cell proliferation required seeding transfected cells at a density of 1000 cells per well in 96-well plates (Corning, NY, U.S.A.). Cell viability was determined by the cell counting kit-8 (CCK-8) system (Vazyme, A311-01) after seeding at 0, 1, 2, 3,4 and 6 days after seeding. In a nutshell, the plate was incubated at 37°C for two hours in dark after adding 10 μl of CCK8 solution to each well. Microplate readers (BioTek, USA) were used to measure absorbance at 450 nm.

For the wound healing assay, cells were inoculated in 6-well plates, a scratch wound was created with a sterile 10 μl pipette tip after cell growth fusion up to 100% and floating cells were removed by washing with 1 × PBS. Photographs were taken under a 100× inverted microscope at 0 h, 24 h, and 48 h after scratching.

Transwell assay was used to measure cell migration. Cells were inoculated into transwell chambers at a density of 2*10^^5^ in 200ul of serum-free medium according to different groups, and then the chambers were placed in 24-well plates with 500ul of medium containing 10% serum. After incubation at 37 °C, 5% CO2 for 24h, the chambers were fixed with 4% paraformaldehyde (Biosharp, BL539A), stained with 0.1% crystal violet stain solution (Solarbio, G1063), and photographed under the microscope.

### Immunohistochemistry

Colorectal adenocarcinoma and adjacent normal (n=10) tissue samples and hepatocellular carcinoma and adjacent normal (n=10) tissue samples were collected at the Xiangya Hospital (Hunan, China). Immunohistochemical staining was carried out according to the kit’s protocol (ZSGB-BIO, PV-9000). Primary antibodies were diluted as follows: TROAP: 1:100 (Proteintech, 13634-1-AP). Local ethics committees approved the research. Two experienced pathologists analyzed eight randomly selected fields in each tissue sample under a microscope to determine the level of protein expression. An expression level was calculated by multiplying the intensity of staining (0 for negative staining, 1 for weak staining, 2 for moderate staining, and 3 for strong staining) by the proportion of immunopositive staining area (0 for ≤5% positive cells, 1 for 6–25%, 2 for 26–50%, 3 for 51–75%, and 4 for ≥76%).

### Statistical analysis

Kaplan-Meier survival curves were drawn using the KM method and survival differences were tested using the log-rank test. For continuous variables, the Student’s t-test was used to determine the statistical difference between any two groups, and ANOVA with the Brown-Forsythe test was used for multiple groups. Categorical variables were compared using the chi-square or Fisher’s exact test. Statistical analyses were performed using the online databases mentioned above or using the R software (version 4.0.3). Statistical significance was determined by P-values > 0.05.

## Results

### Profile of TROAP expression in pan-cancer and its correlation with clinicopathological characteristics

**Figure 1:**
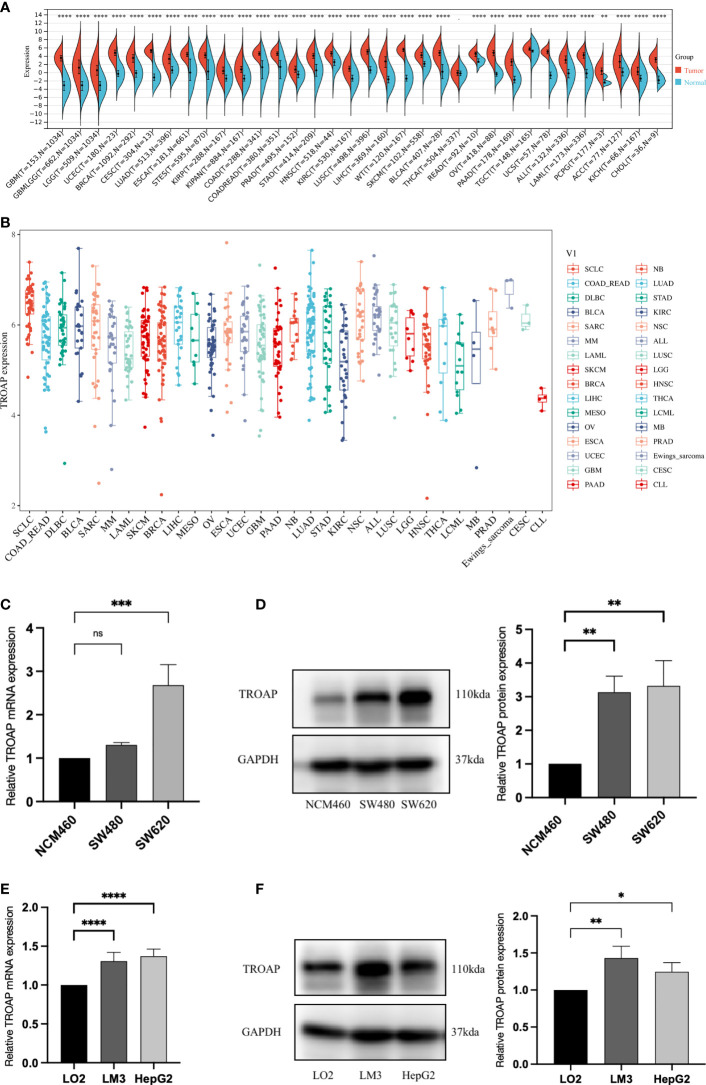
Differential transcriptional expression of TROAP. **(A)** TROAP expression in tumor tissues and paired normal tissues in various cancers according to the RNA sequencing data from the TCGA database *via* the SangerBox online tool. **(B)** The transcriptional expression of TROAP in different cancer cell lines was analyzed by the CCLE database. **(C)** The results of qRT-PCR showed that the mRNA levels of TROAP in colorectal cancer (CRC) cells (SW480, SW620) were significantly elevated compared with normal colorectal mucosa cells (NCM460). **(D)** The results of Western blotting showed that the protein expression level of TROAP was upregulated in CRC cells compared to the NCM460 cells. **(E)** The results of qRT-PCR demonstrated that the mRNA levels of TROAP were up-regulated in hepatocellular carcinoma (HCC) cells (HCC-LM3, HepG2) compared to the normal liver cells (LO2). **(F)** The protein expression levels of TROAP in LO2 cells and HCC cells (Western blotting). TROAP protein was much higher in HCC cell lines than that in LO2 cells. All *: P < 0.05, **: P < 0.01, ***:P < 0.001, ****:P < 0.0001, and ns: no significance.

**Figure 2:**
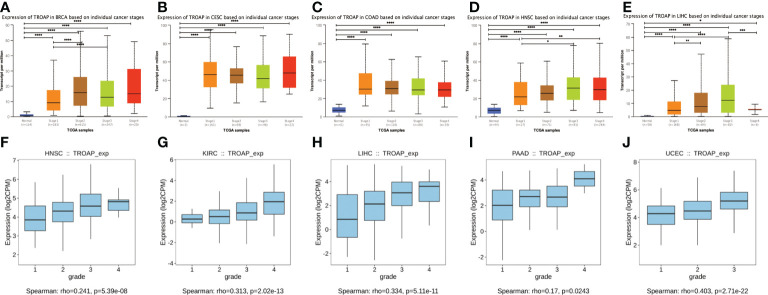
The transcriptional expression of TROAP in pan-cancer stratified by stage and grade, respectively. **(A–E)** The association between TROAP transcriptional level and various tumor stages in breast invasive carcinoma(BRCA), cervical squamous cell carcinoma and endocervical adenocarcinoma (CESC), colon adenocarcinoma (COAD), head and neck squamous cell carcinoma (HNSC) and liver hepatocellular carcinoma(LIHC) using TISIDB database; **(F–J)** The correlation between the transcriptional level of TROAP and different tumor grades HNSC, kidney renal clear cell carcinoma (KIRC), LIHC, pancreatic adenocarcinoma (PAAD), and uterine corpus endometrial carcinoma (UCEC) using UALCAN online tool. All *: P < 0.05; **:P < 0.01; ***: P < 0.001; and **** P < 0.0001.

**Figure 3:**
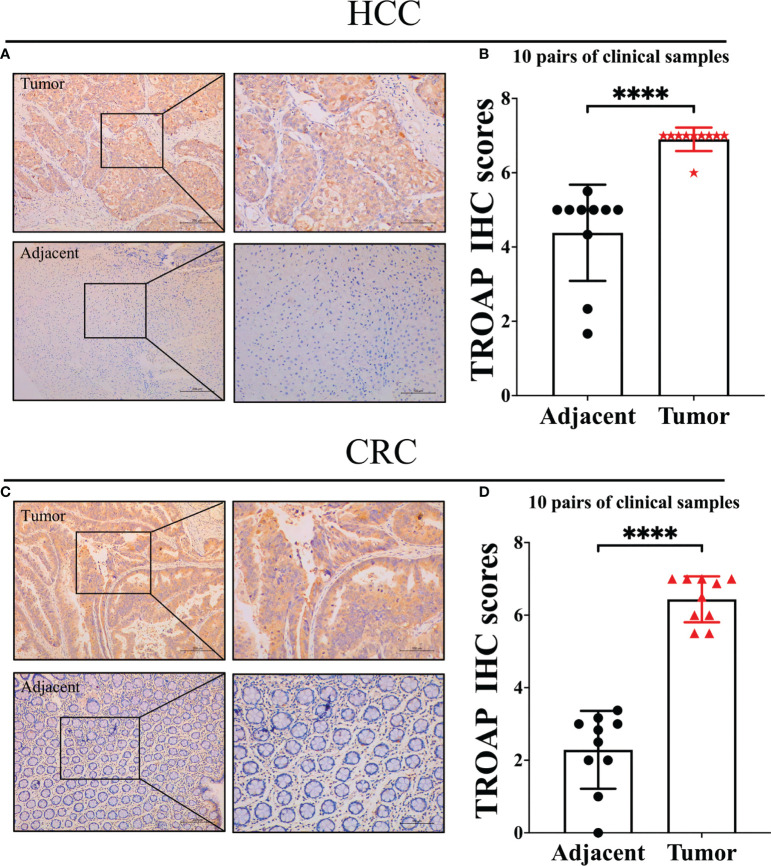
Representative immunohistochemical staining in tumor and tumor-adjacent normal tissue. **(A)** TROAP staining score was statistically higher in HCC tissues compared to adjacent tissues; **(B)** Statistical analysis of immunohistochemical staining intensity of HCC group and adjacent group(n=10, p<0.0001); **(C)** TROAP staining score was statistically higher in CRC tissues compared to adjacent tissues; **(D)** Statistical analysis of immunohistochemical staining intensity of CRC group and adjacent group (n=10, p<0.0001). ****:P < 0.0001.

TROAP mRNA levels were remarkably higher in 33 human cancers than in adjacent tissues (**Figure 1A**). TROAP expression increased in almost all cancer cell lines, with the highest level observed in small cell lung carcinoma and the lowest expression in chronic lymphocytic leukemia (**Figure 1B**). Then, we verified that TROAP mRNA and protein levels in colorectal cancer (CRC) and hepatocellular carcinoma (HCC) cells were significantly higher than those in the corresponding normal cells based on qRT-PCR and Western blot analysis, consistent with the above-mentioned database results (**Figures 1C–F**). Moreover, TROAP expression is widely found in extensive normal tissues, among which 721_B_lymphoblasts and endothelial cells (CD105+, CD34+) and thymus and testis tissues showed a higher TROAP expression level (**Figure S1**). The upregulation of TROAP expression in cancer tissues and immune cells may be involved in immune regulation. Moreover, TROAP expression was significantly associated with a specific patient population stratified by clinical stage, as shown by an increase in TROAP expression with the escalating clinical stage (**Figures 2A–E**; **Figure S2**). TROAP expression was significantly correlated with tumor grade in the five cancer types (**Figures 2F–J**). Furthermore, the Human Protein Atlas (HPA) data showed that the immunohistochemistry (IHC) staining of tumor samples was higher than that of the corresponding adjacent normal samples in various cancers (**Figure S3**). And our own IHC results further confirmed that the expression level of TROAP was significantly higher in HCC and CRC tissues than in adjacent tissues, and the difference was statistically significant (**Figure 3**).

### Prognostic value of TROAP expression in pan-cancer

To further evaluate the prognostic value of TROAP expression in pan-cancer, a univariate Cox regression analysis was conducted to investigate the relationship between TROAP expression and various survival outcomes for each cancer, including overall survival (OS), disease-free survival (DFS), disease-specific survival (DSS), and progression-free interval (PFI). In the OS analysis, high TROAP expression was a risk factor for 15 tumors. However, it appears to be a protective factor against thymoma (THYM) and ovarian serous cystadenocarcinoma (**Figure S4A**). Furthermore, in the GEPIA2 database, Kaplan–Meier (K-M) survival analysis indicated that poor OS was predicted by high TROAP expression, while low TROAP expression was correlated with shortened OS in THYM. This was roughly in line with the results of the Cox regression analysis (**Figures S5A–K**). Moreover, TROAP expression was apparently associated with poor prognosis and DFS in various cancers (**Figure S4B**). Consistently, the K-M survival curve indicated shorter DFS in patients with elevated TROAP expression (**Figures S5L–W**). The forest plots showed that high TROAP expression predicted poor DSS in 15 cancers and poor DFI in 18 cancers (**Figures S4C, D**). Additionally, survival analysis using the PrognoScan database demonstrated that high TROAP expression predicted poor survival (**Figure S6**).

### TROAP-induced malignant phenotype and tumorigenesis *via* activating PI3K/Akt/GSK-3β signaling of HCC and CRC cells *in vitro*

**Figure 4:**
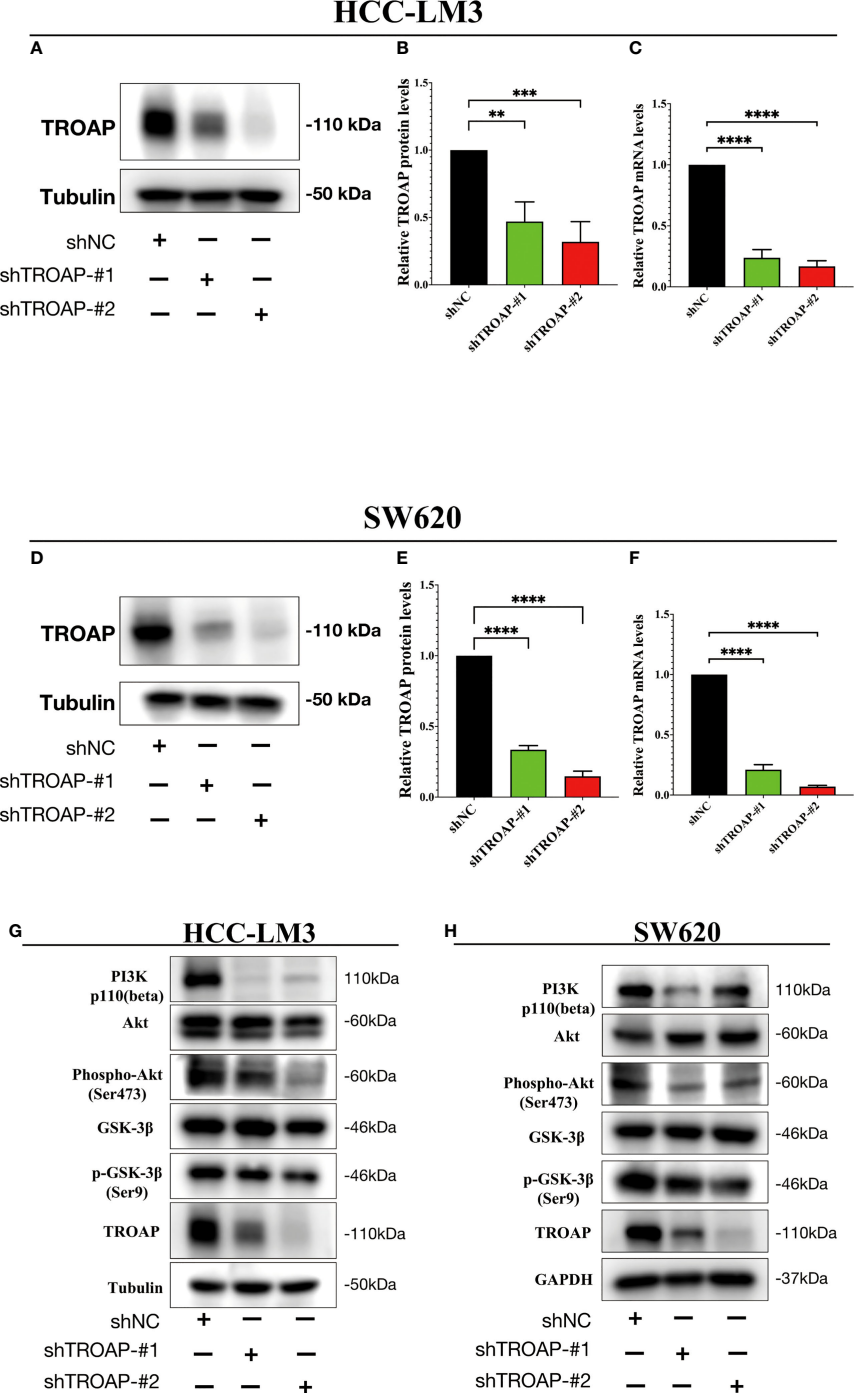
TROAP-induced malignant phenotype *via* activating the PI3K/Akt/GSK3β signaling in HCC and CRC cells. **(A, B, D, E)** The downregulation of TROAP in HCC-LM3 **(A)** and SW620 **(D)** cells after lentivirus-mediated silence of TROAP were confirmed by Western blot. The statistics were shown in histograms **(B**, **E)**. **(C, F)** TROAP knockdown in HCC-LM3 and SW620 cells were verified by qRT-PCR. **(G, H)** Western blot analysis showing the expression of PI3K/Akt/GSK3β signaling-related proteins after silencing TROAP in HCC-LM3and SW620 cells. All **:P < 0.01, ***:P < 0.001 and ****:P < 0.0001.

**Figure 5:**
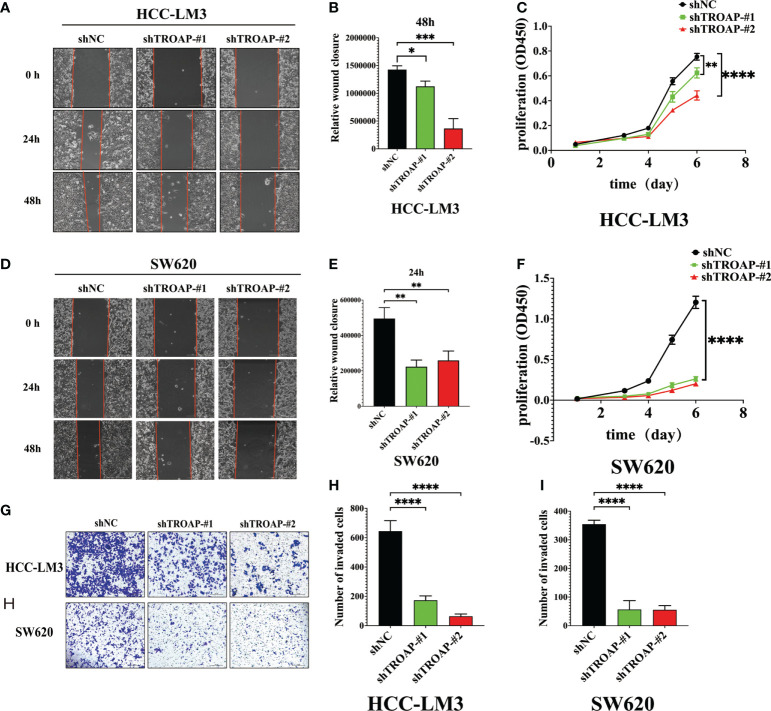
Downregulation of TROAP attenuates the malignant proliferation and migration of HCC and SW620 cells *in vitro*. **(A, B, D, E)** Wound healing assay, images of the HCC-LM3 and SW620 cells after scratching 0h, 24h, and 48h are shown in **(A, D)**, and percentages of wound closure were shown in **(B, E)**; **(C, F)** The proliferation rate of HCC-LM3 and SW620 cells after lentivirus-mediated silence of TROAP determined by CCK8 assay; **(G-J)** Transwell assay to access the effects of TROAP knockdown on cell migration in HCC-LM3 and SW620 cells, quantification graphs were shown in **(I, J)**. In panels **(B–J)**, data are indicated as mean ± SD; two-sided Student’s t-test; *P < 0.05; **P < 0.01; ***:P < 0.001 and ****:P < 0.0001.

To investigate the proliferation and migration role of TROAP, two shRNAs targeting TROAP (shTROAP) were stably transfected into HCC-LM3(hepatocellular carcinoma cells) and SW620 (colorectal cancer) cells that highly expressed TROAP, respectively (**Figures 4A–F**). The cell proliferation was analyzed by CCK8 assay, which demonstrated the decreased proliferation rate of HCC-LM3 and SW620 cells after the knockdown of TROAP expression. And transwell and wound healing assays were used to investigate TROAP’s role in cell migration. Findings demonstrated that knocking down TROAP impaired the migration ability of HCC-LM3 and SW620 cells (p < 0.05, **Figure 5**). Furthermore, the molecular mechanisms underlying TROAP-induced oncogenic phenotype were also examined. The results of western blot analysis illustrated that TROAP silence could significantly downregulate the level of downstream target genes of PI3K/Akt/GSK-3β signaling, such as PI3K, p-AKT, and p-GSK3B in HCC-LM3 and SW620 cells compared to control (p < 0.05, **Figures 4G, H**). As a result, our results manifested that tumorigenesis might be induced through activation of the PI3K/Akt/GSK-3β signaling pathway.

### Correlation between TROAP expression and genetic and post-transcriptional alteration in pan-cancer

**Figure 6:**
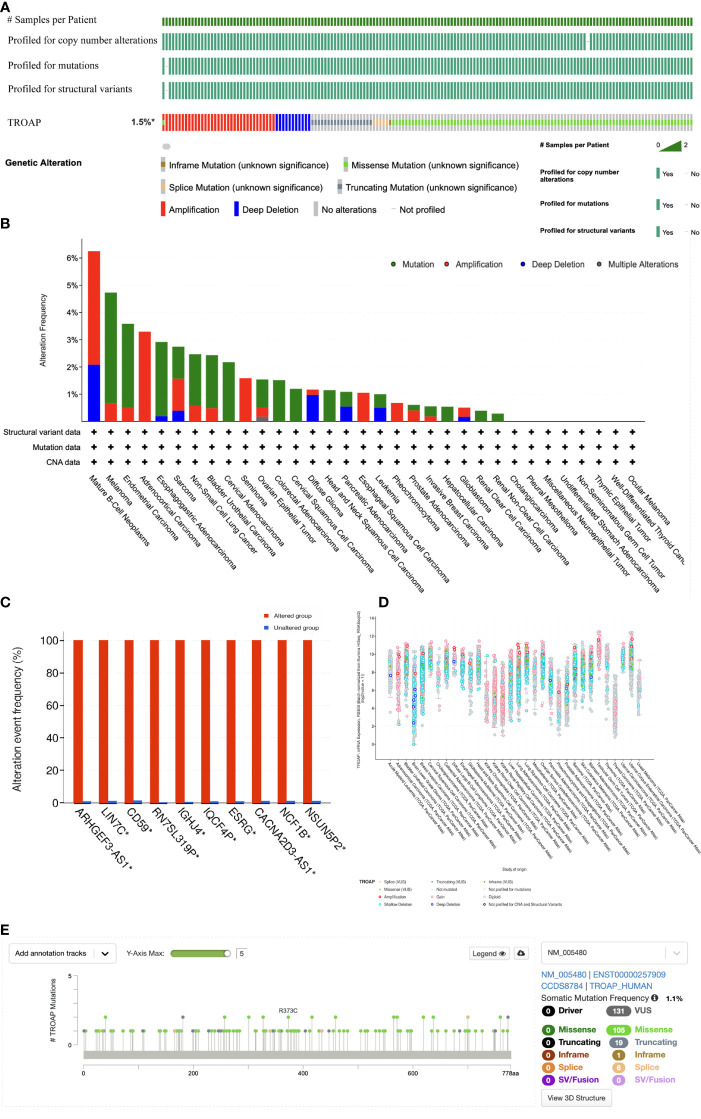
The genetic alteration profiles of TROAP in pan-cancer from TCGA database analyzed by cBioPortal tool. **(A)** Summary of the alteration on TROAP gene in pan-cancer: The TROAP gene was altered which accounted for only 1.5% across 10,967 samples *via* using the cBioPortal database. **(B)** The alteration frequency of TROAP including structural variant, mutations, and copy-number alterations data in TCGA pan-cancer datasets. **(C)** The analysis of gene mutation co-occurrence comparing the altered group and unaltered group of TROAP. **(D)** The major types of TROAP gene alterations in various cancers. **(E)** The location of main mutation types and corresponding numbers in the site of TROAP amino acid sequence.

**Figure 7:**
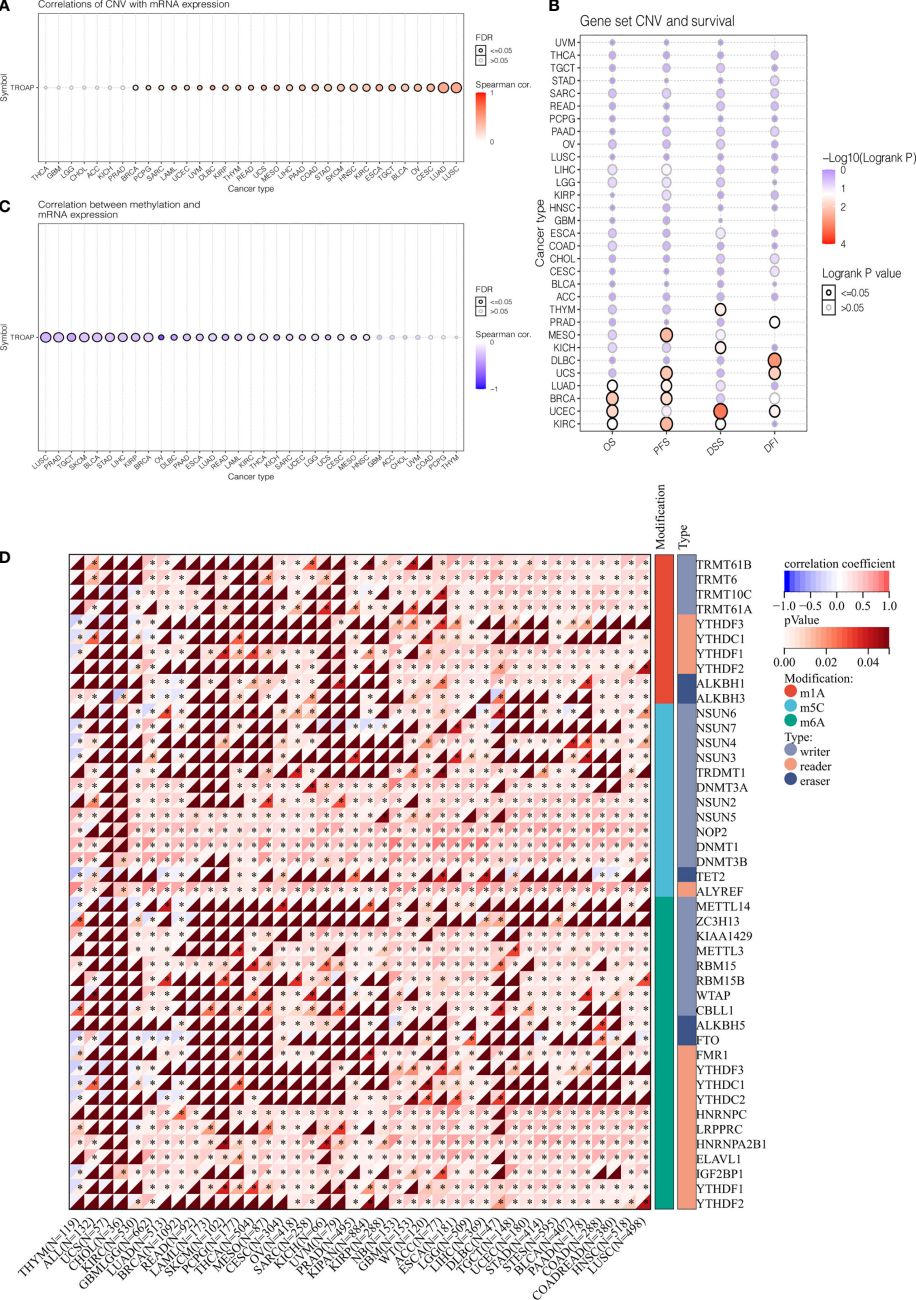
The correlation of TROAP transcriptional expression and its post-transcriptional modification in pan-cancer. **(A)** The association of TROAP expression and copy number variation (CNV). **(B)** The association between TROAP gene set CNV and survival possibility in 31 cancer types. **(C)** The correlation between TROAP expression and gene promoter methylation. **(D)** The correlation between TROAP expression and the gene markers of three classes of RNA modified patterns (m1A, m5C, and m6A). All *: P < 0.05.

The TROAP gene was altered, accounting for only 1.5% across 10,967 samples, and the most frequent alteration was a mutation, which occurred in 17 cancer types (**Figures 6A, B, E**). Co-occurrence of ARHGEF3-AS1 and LIN7C alterations was observed with TROAP alterations (**Figure 6C**). Additionally, mutation of the TROAP gene was intimately correlated with TROAP transcriptional expression in pan-cancer (**Figure 6D**). Then, the GSCA database was used to indicate a substantial positive connection between the copy number variation (CNV) of the TROAP gene and its mRNA expression levels in 26 cancers (**Figure 7A**). TROAP gene CNV was positively correlated with OS in kidney renal clear cell carcinoma (KIRC), uterine corpus endometrial carcinoma (UCEC), breast invasive carcinoma(BRCA), and lung adenocarcinoma (LUAD) (**Figure 7B**). A negative relationship exists between the methylation of the TROAP gene and its mRNA levels in most cancers (**Figure 7C**). Moreover, the SangerBox was used to analyze the correlation between TROAP expression and gene markers included in the three classes of RNA modification, namely, N6-methyladenosine (m6A), N1-methyladenosine (m1A), and 5-methylcytidine (m5C). The results showed a strong positive correlation between TROAP and most gene markers relevant to RNA modification in various cancers (**Figure 7D**).

**Figure 8:**
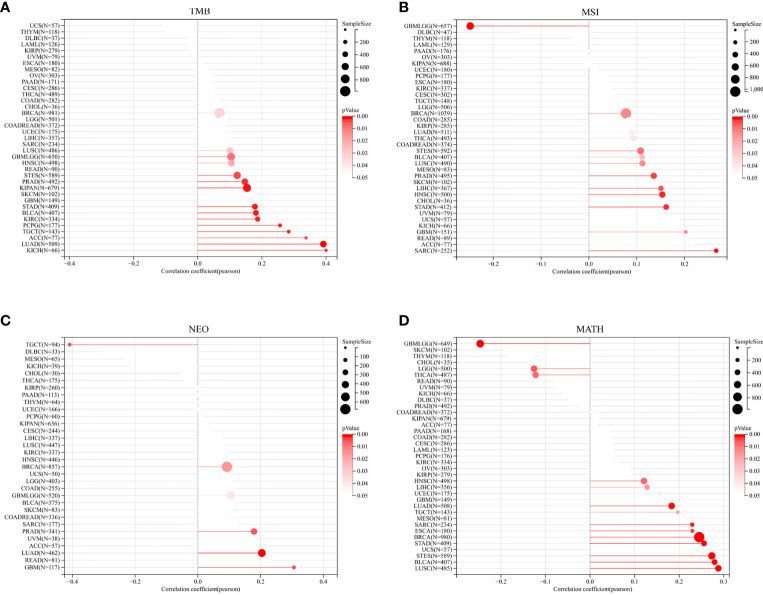
The relationship between the transcriptional level of TROAP and the genetic mutation in pan-cancer. **(A)** tumor mutational burden (TMB). **(B)** microsatellite instability (MSI). **(C)** neoantigen (NEO). **(D)** mutant-allele tumor heterogeneity (MATH).

Subsequently, TROAP expression showed a significant positive association with TMB in 16 of 37 cancers, among which kidney chromophobe (KICH) had the highest correlation coefficient and breast invasive carcinoma had the lowest correlation coefficient (**Figure 8A**). Furthermore, TROAP expression was significantly related to MSI in 14 cancers, NEOs in six cancers, and MATH in 16 cancers (**Figures 8B–D**). Therefore, tumorigenesis and cancer progression may be influenced by TROAP genomic alteration and differential expression in cancer tissues.

### Relationship between TROAP expression and immune types and tumor-infiltrating lymphocytes (TILs) in human cancers

**Figure 9:**
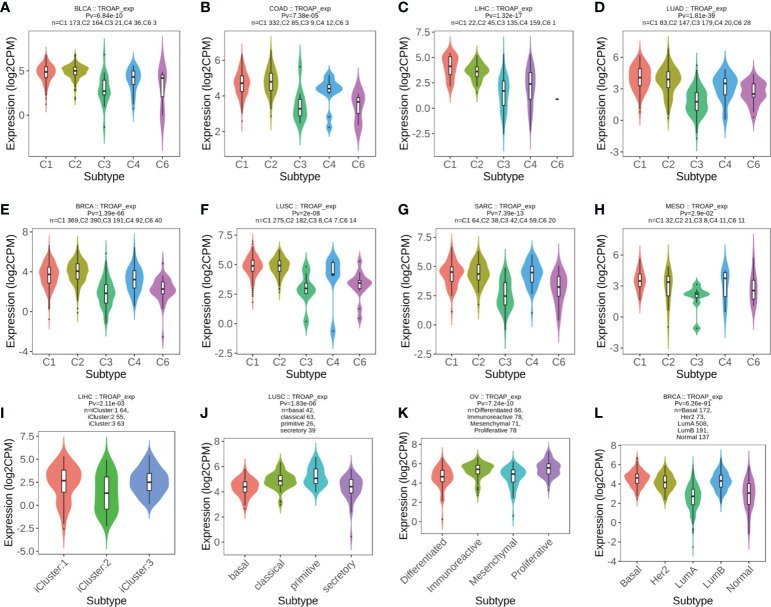
**(A–H)** The correlation of TROAP transcriptional expression and immune subtypes in **(A)** bladder urothelial carcinoma (BLCA), **(B)** COAD, **(C)** LIHC, **(D)** lung adenocarcinoma (LUAD), **(E)** BRCA, **(F)** lung squamous cell carcinoma (LUSC), **(G)** sarcoma (SARC), and **(H)** mesothelioma (MESO). **(I–L)** The relationship between TROAP transcriptional expression and molecular subtypes in **(I)** LIHC, **(J)** LUSC, **(K)** ovarian serous cystadenocarcinoma (OV), and **(L)** BRCA. C1 (wound healing); C2 (IFN-gamma dominant); C3 (inflammatory); C4 (lymphocyte depleted); C5 (immunologically quiet); C6 (TGF-b dominant).

The TISIDB database revealed significant correlations between TROAP expression levels and immune subtypes in 20 cancers. TROAP was expressed at low levels in the C3 (inflammatory) type in most of the 20 cancers (**Figures 9A–H**, **Figures S7A–L**). Furthermore, we found a significant association between TROAP expression and molecular subtypes in various cancers (**Figures 9I–L**, **Figures S7M-T**). The above-mentioned results suggest that TROAP expression differs in various cancers’ immune and molecular subtypes. ESTIMATE is an algorithm for predicting tumor purity in the TME, including stromal, immune, and estimate scores (34). Subsequently, we explored the associations between TROAP expression and three types of ESTIMATES. The estimated score showed strong positive correlations with TROAP expression in six cancers and negative correlations in 23 cancers. Among the 44 tumor types, TROAP was significantly correlated with stromal scores in 30 cancer types and immune scores in 27 cancers (**Table 1**).

**Table 1:** Correlation analysis of TROAP expression with ESTIMATE scores in pan-cancers according the RNA sequencing data from TCGA database.

Cancer	Stromal Score		Immune Score		ESTIMATE Score	
	pearson_R	P-value	pearson_R	P-value	pearson_R	P-value
TCGA-GBM (N=152)	-0.38	***	-0.43	***	-0.43	***
TCGA-GBMLGG (N=656)	0.28	***	0.26	***	0.28	***
TCGA-LGG (N=504)	0.07	ns	0.12	**	0.10	*
TCGA-UCEC (N=178)	-0.32	***	-0.26	***	-0.31	***
TARGET-LAML (N=142)	0.09	ns	0.11	ns	0.11	ns
TCGA-BRCA (N=1077)	-0.34	***	-0.02	ns	-0.18	***
TCGA-CESC (N=291)	-0.30	***	-0.30	***	-0.34	***
TCGA-LUAD (N=500)	-0.25	***	-0.21	***	-0.25	***
TCGA-ESCA (N=181)	-0.23	**	-0.33	***	-0.31	***
TCGA-STES (N=569)	-0.41	***	-0.34	***	-0.41	***
TCGA-SARC (N=258)	-0.18	**	-0.10	ns	-0.14	*
TCGA-KIRP (N=285)	0.00	ns	0.00	ns	0.00	ns
TCGA-KIPAN (N=878)	0.14	***	0.20	***	0.18	***
TCGA-COAD (N=282)	-0.33	***	-0.18	**	-0.27	***
TCGA-COADREAD (N=373)	-0.31	***	-0.17	***	-0.26	***
TCGA-PRAD (N=495)	0.04	ns	0.07	ns	0.06	ns
TCGA-STAD (N=388)	-0.44	***	-0.30	***	-0.40	***
TCGA-HNSC (N=517)	-0.29	***	-0.18	***	-0.26	***
TCGA-KIRC (N=528)	0.01	ns	0.23	***	0.15	***
TCGA-LUSC (N=491)	-0.47	***	-0.38	***	-0.45	***
TCGA-THYM (N=118)	-0.24	*	0.45	***	0.21	*
TCGA-LIHC (N=363)	-0.26	***	0.05	ns	-0.09	ns
TARGET-WT (N=80)	-0.22	*	-0.33	**	-0.30	**
TCGA-SKCM-P (N=101)	-0.45	***	-0.24	*	-0.35	***
TCGA-SKCM (N=452)	-0.25	***	-0.18	***	-0.22	***
TCGA-BLCA (N=405)	-0.12	*	-0.05	ns	-0.09	ns
TCGA-SKCM-M (N=351)	-0.20	***	-0.16	**	-0.19	***
TCGA-THCA (N=503)	0.31	***	0.36	***	0.36	***
TARGET-NB (N=153)	-0.41	***	-0.25	**	-0.34	***
TCGA-MESO (N=85)	0.04	ns	-0.09	ns	-0.04	ns
TCGA-READ (N=91)	-0.27	*	-0.18	ns	-0.24	*
TCGA-OV (N=416)	-0.21	***	-0.15	**	-0.20	***
TCGA-UVM (N=79)	-0.07	ns	-0.04	ns	-0.06	ns
TCGA-PAAD (N=177)	-0.11	ns	-0.04	ns	-0.08	ns
TCGA-TGCT (N=132)	-0.45	***	-0.30	***	-0.43	***
TCGA-UCS (N=56)	0.00	ns	-0.01	ns	0.00	ns
TCGA-LAML (N=214)	0.26	***	-0.07	ns	0.08	ns
TARGET-ALL (N=86)	-0.15	ns	-0.03	ns	-0.08	ns
TCGA-PCPG (N=177)	-0.03	ns	-0.08	ns	-0.06	ns
TCGA-ACC (N=77)	-0.20	ns	-0.24	*	-0.23	*
TARGET-ALL-R (N=99)	-0.29	**	-0.36	***	-0.36	***
TCGA-DLBC (N=46)	-0.02	ns	-0.30	*	-0.20	ns
TCGA-KICH (N=65)	-0.04	ns	-0.13	ns	-0.09	ns
TCGA-CHOL (N=36)	-0.38	*	-0.16	ns	-0.25	ns

ESTIMATE: estimation of stromal and immune cells in malignant tumor tissues using expression data. Estimate Score included stromal score, immune score and estimate score. All *: P < 0.05, **: P < 0.01, ***:P < 0.001, and ns: no significance.

**Figure 10:**
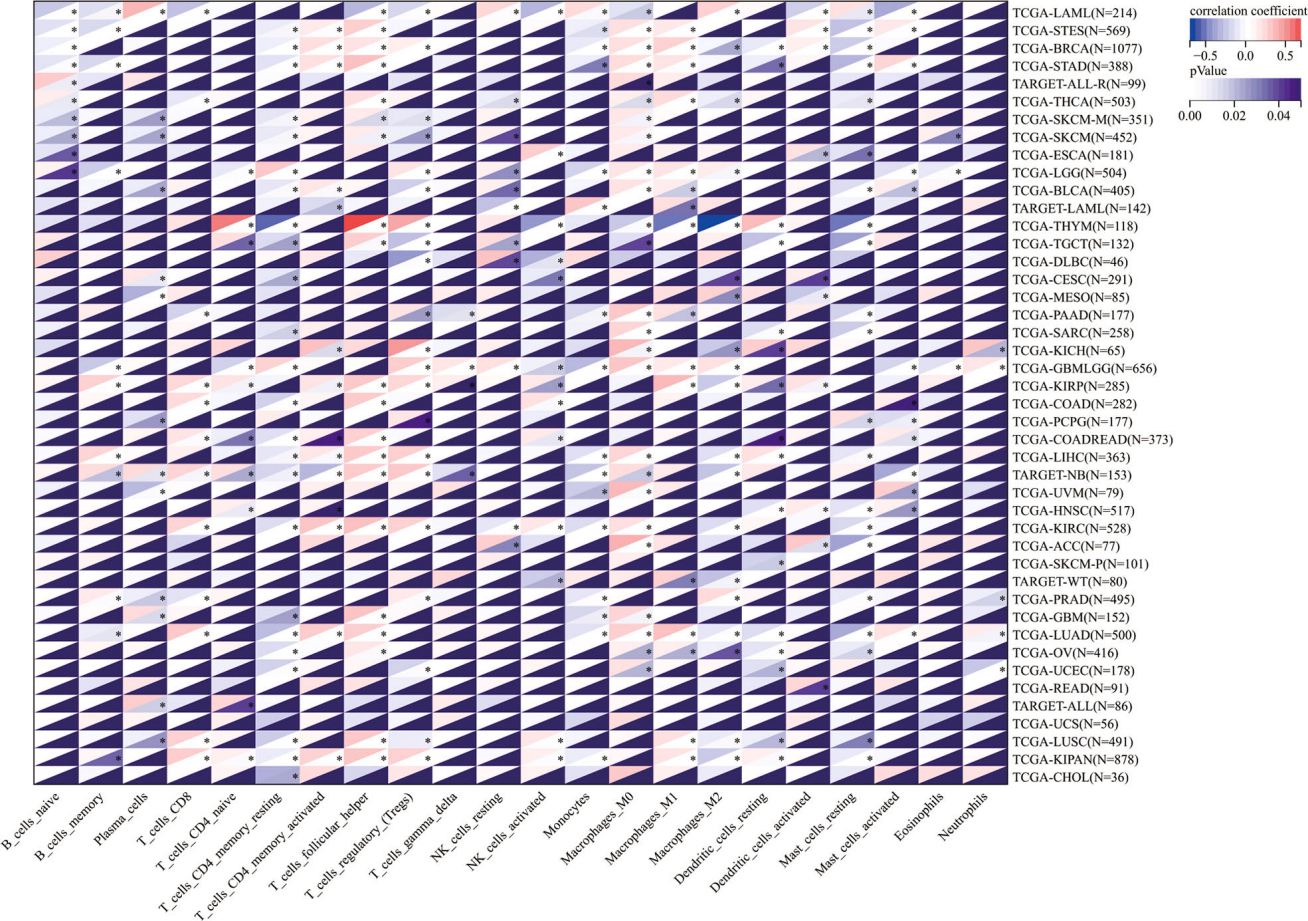
The correlation of TROAP transcriptional level and the infiltration of 22 immune cell subtypes in pan-cancer. All *: P < 0.05.

Then, the correlation between TROAP expression and infiltration of six immune cell subtypes of B cells, CD4 and CD8 T cells, dendritic, macrophages, and neutrophils cells was investigated, demonstrating that TROAP expression is significantly positively correlated to TILs in several cancers, such as liver hepatocellular carcinoma (LIHC) as well as kidney renal clear cell carcinoma (KIRC) (**Figure S8**). Previous studies have indicated that TILs can be used as independent prognostic predictors for cancer (35). Therefore, we further examined the association between TROAP levels and infiltrating levels of 22 immune cell types in pan-cancer derived from CIBERSORT (36), showing that TROAP expression was significantly related to various immune cell subtypes. For instance, its expression is negatively correlated with T cell CD4 memory resting in 24 cancers and positively correlated with T_cells_regulatory_(Tregs) in 13 cancers (**Figure 10**). Further single-cell analysis of tumor immunity using the TISCH database revealed a notably positive correlation between TROAP and Proliferating T cells in 26 tumors (**Figure S10**). Generally, the above-mentioned results indicated that TROAP expression influenced TILs in pan-cancer.

### Co-expression relevance of TROAP and immunomodulation-related genes in TME in pan-cancer

**Figure 11:**
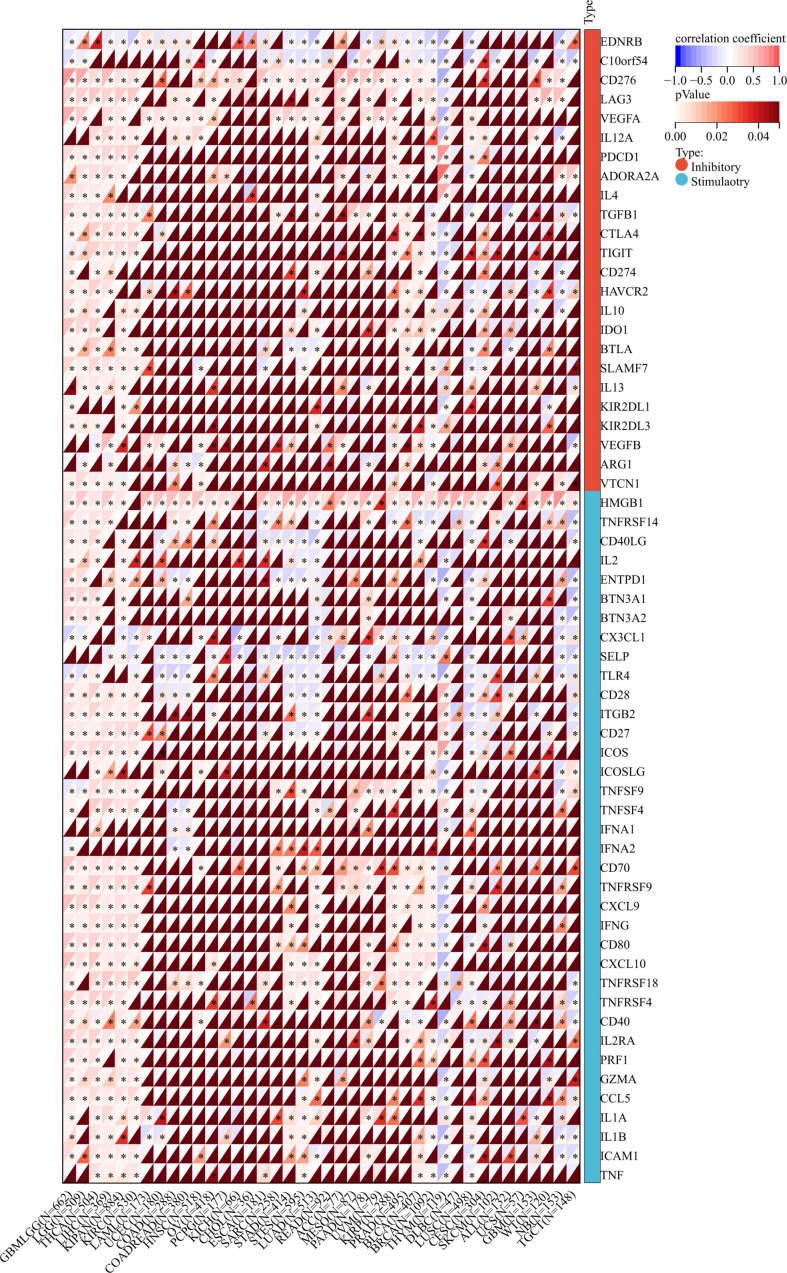
The relationship between TROAP expression and immune regulatory gene markers in pan-cancer. All *:P < 0.05.

Immune checkpoints (ICs) are ligands that bind to inhibitory receptors on immune cells, which downregulate the T-cell-mediated immune response (37). Then, a correlation analysis of TROAP expression and 60 immune checkpoint biomarkers was conducted, including 24 inhibitory and 36 stimulatory molecules (38). As for immune inhibitors, TROAP expression was positively linked to CD276 in 29 tumors and LAG3 in 21 tumors but negatively associated with EDNRB in 25 tumors and C10orf54 in 22 tumors. Regarding immune stimulators, TROAP expression was positively associated with HMGB2 in most tumors but negatively correlated with SELP in 24 tumors and TLR4 in 22 tumors (**Figure 11**). A novel immunotherapy target, TROAP may act by coordinating immune checkpoint gene activity in multiple signaling pathways.

**Figure 12:**
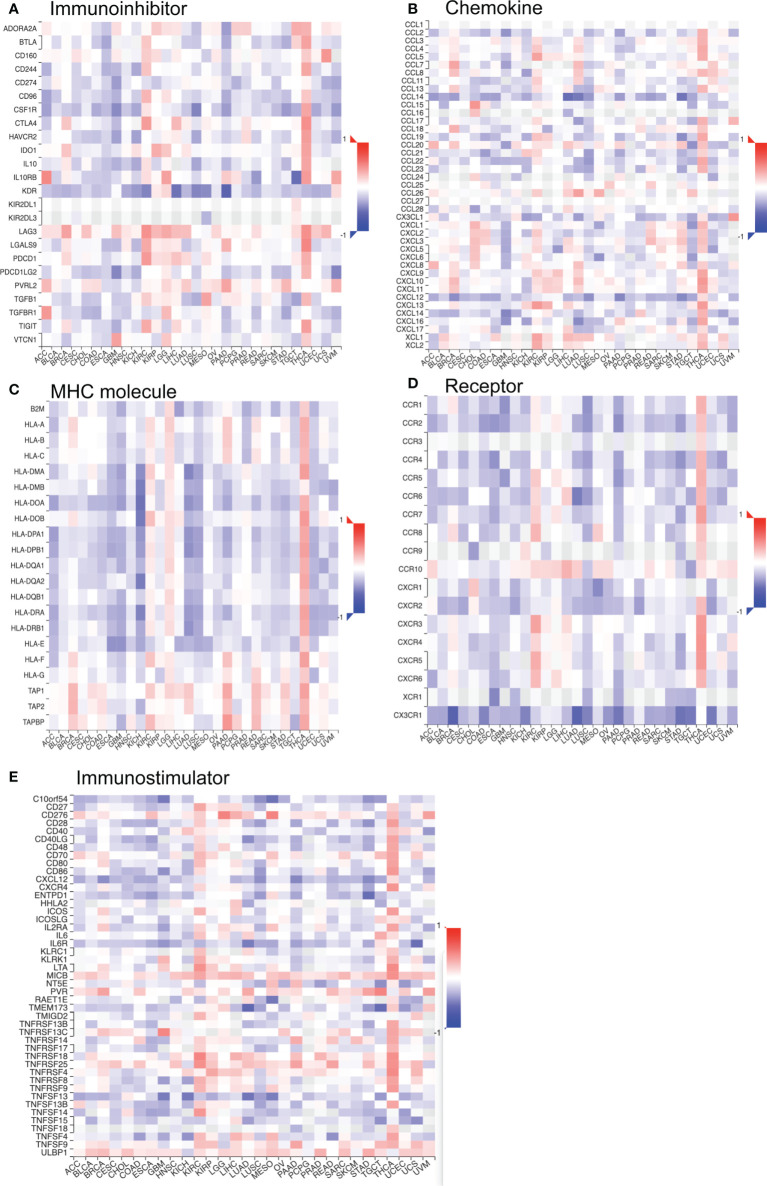
The co-expression correlation of TROAP transcriptional expression and gene markers of specific immune-regulated gene sets in pan-cancer. **(A)** Immunoinhibitor. **(B)** Chemokine. **(C)** Major histocompatibility complex (MHC) Molecules. **(D)** Chemokine receptor. **(E)** Immunostimulator. Red and blue color represented the positive and negative correlation, respectively. And the degree of color from dark to light presented the correlation gradually changed from strong to weak.

In addition, we investigated the relationship between TROAP expression and immune-related gene sets in 33 tumors, including chemokine, receptor, immunostimulator, immunoinhibitor, and genes encoding major histocompatibility complex (MHC) molecules. The majority of immune-related genes were positively associated with TROAP expression in glioma, brain lower-grade glioma (LGG), pancreatic adenocarcinoma (PAAD), and thyroid carcinoma (THCA), LIHC, pan-kidney cohort, and KIRC. Conversely, most gene biomarkers were negatively correlated with TROAP expression in THYM, testicular germ cell tumors, glioblastoma multiforme, NB, and lung squamous cell carcinoma (**Figure 12**). As a result of these results, TROAP plays a crucial role in regulating the expression and function of gene markers related to TME in cancers.

### Association of TROAP expression and drug sensitivity in human cancers

**Figure 13:**
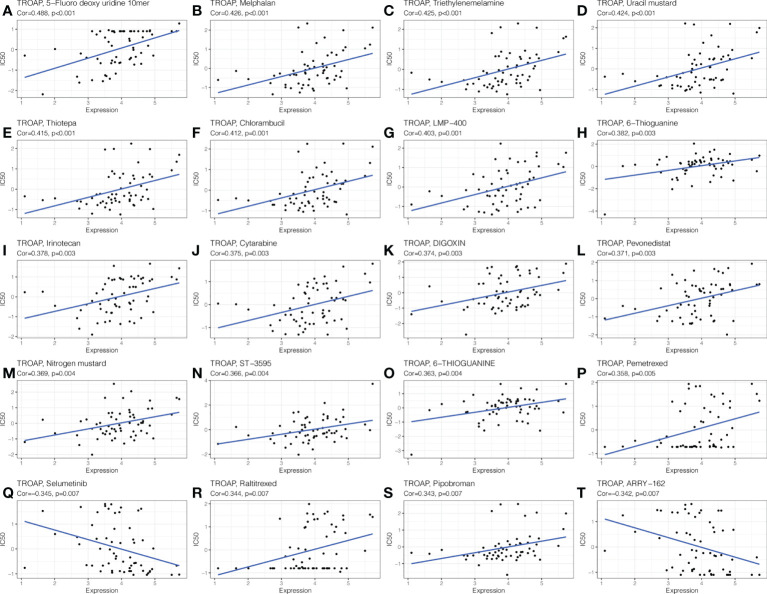
The drug sensitivity analysis of TROAP expressed level and various chemical drugs in pan-cancer. The TROAP expression was positively associated with drug sensitivity of **(A)** 5−Fluoro deoxy uridine 10mer, **(B)** Melphalan, **(C)** Triethylenemelamine, **(D)** Uracil mustard, **(E)** Thiotepa, **(F)** Chlorambucil, **(G)** LMP−400, **(H)** 6−Thioguanine, **(I)** Irinotecan, **(J)** Cytarabine, **(K)** DIGOXIN, **(L)** Pevonedistat, **(M)** Nitrogen mustard, **(N)** ST−3595, **(O)** 6−THIOGUANINE, **(P)** Pemetrexed, **(R)** Raltitrexed, and **(S)** Pipobroman. The TROAP expression was negatively associated with drug sensitivity of **(Q)** Selumetinib and **(T)** ARRY−162. The *X*-axis represents the TROAP expression level and *Y*-axis represents the value of IC50.

The CellMiner database indicated drug sensitivity and TROAP expression were positively correlated in patients treated with 18 drugs, such as 5-fluoro deoxyuridine 10mer, melphalan, thiotepa, and chlorambucil. There is a negative correlation between TROAP expression and anticancer drug treatment with selumetinib and ARRY-162 (**Figure 13**). These drugs are used for chemotherapy, endocrine therapy, and targeted therapy of tumors. Further, the CTRP database found that drug sensitivity was negatively correlated with TROAP expression, except for ML210, PD318088, selumetinib, and trametinib, while they were positively correlated in the GDSC database (**Figure S9**).

## Discussion

TROAP has been shown to participate in mitosis and cell cycle progression in previous studies and is involved in the malignant biological behavior of a variety of tumors (19, 39). In prostate cancer, the knockdown of TROAP reduced cell proliferation ability and induced cell cycle arrest and apoptosis (39), whereas TROAP overexpression led to G1/S phase arrest in LIHC (40). Although TROAP has been recognized as an unfavorable factor in several cancers, the molecular function and precise mechanism of TROAP in tumorigenesis and proliferation of many cancers have yet to be elucidated, and no research has focused on its impact on pan-cancer. In this study, an extensive examination of the role of TROAP in a variety of cancers was undertaken with the aim of demonstrating a comprehensive workflow for pan-cancer analysis.

TROAP expression was significantly higher in most cancers, which was in accordance with previous research on lung cancer and LIHC (17, 18), and with our confirmed results using qRT-PCR and Western blot analysis. In normal cells, TROAP expression levels were higher in immune cells, suggesting that TROAP may be involved in immune regulation. The expression of TROAP increased as the clinical and tumor stages increased. TROAP promotes oncogenesis and cancer progression, according to these results. As in previous studies, patients with high TROAP expression had a poorer prognosis in lung cancer, LIHC (18, 41), and KIRC (42), and in many other cancers, such as LGG, mesothelioma, and PAAD, high TROAP expression indicated worse prognosis, confirming that TROAP is a biomarker that may be useful for predicting cancer outcomes.

TROAP may be a valuable prognostic indicator for tumors, based on these findings. We performed follow-up experiments to determine whether TROAP influences cancer cell biological behavior. Migration and proliferation are malignant phenotypes of cancer cells. Through interference with the TROAP protein, the CCK8 assay showed a significant decrease in cell proliferation activity. In transwell and wound healing assays, TROAP knockdown reduced tumor cell migration ability. Therefore, the high expression of TROAP in tumors may be an important protein affecting the malignant progression of tumors. PI3K/Akt signaling pathway, as one of the important intracellular signal transduction pathways, is a key player in tumorigenesis and development, it has a close relationship with tumor cell therapy resistance, proliferation and apoptosis, cycle regulation, invasion, and metastasis (43, 44). To further elucidate the molecular mechanism of TROAP-induced tumor phenotype, this study assessed the expression levels of proteins related to the PI3K/Akt/GSK3β signaling pathway. The results showed that the phosphorylation levels of PI3K, Akt, and GSK3β were significantly decreased after TROAP knockdown in HCC-LM3 and SW620 cells, without affecting their total protein levels, indicating that the PI3K/Akt/GSK3β pathway was inhibited. Therefore, Tumor progression may be facilitated by TROAP activating the PI3K/Akt/GSK3β signaling pathway.

RNA modification is orchestrated by the coordinated actions of a series of writer, reader, and eraser proteins. It is a key epigenetic process in regulating post-transcriptional gene expression. The most common of these are m6A, m1A, and m5C modifications (45). In eukaryotic cells, m6A is the most prevalent internal modification of RNA that plays a crucial role in multiple tumorigeneses (46), and CNVs of m5C regulatory genes are significantly correlated across many cancer types (47). We found that the expression of TROAP was positively correlated with most proteins associated with m6A, m1A, and m5C modifications. Subsequently, DNA methylation is an epigenetic mechanism involving chemical modifications of DNA that can alter genetic performance without altering the DNA sequence (48). In cancer patients, levels of TROAP methylation could be used as a biomarker of prognosis based on TROAP expression and DNA methylation. Therefore, based on the abovementioned relationship between TROAP expression and its methylation level, we hypothesized that TROAP may be involved in tumorigenesis at the epigenetic level, thereby promoting tumor progression.

As precision medicine develops, MSI and TMB can serve as promising pan-cancer biomarkers (7). Tumor cells express a high level of neoantigens, which are immunogenic and heterogeneous in nature (8, 49). They are new biomarkers for the evaluation of the therapeutic efficacy of ICIs that augment adaptive immunity (9). TROAP expression was significantly associated with neoantigens, TMB and MSI. TMB and MSI of cancer are affected by TROAP expression, which affects treatment response to immune checkpoint suppression. Moreover, the mutation-allele fraction values of tumor-specific variant sites can be represented effectively by MATH. Hence, the larger the MATH value, the higher the tumor heterogeneity. We observed that TROAP expression was correlated with MATH in 16 tumors, with a significant positive correlation in 13 tumors. Therefore, this may indicate that the TROAP expression level affects the MATH of cancers and the patient’s OS.

Then, TROAP is significantly disparately expressed in diverse immune and molecular subtypes of most tumors and may be a hopefully pan-cancer diagnostic biomarker involved in immune regulation. Also, ESTIMATE was used as a metric to evaluate cancer patients’ prognoses (50). Most human cancer types also showed negative correlations between TROAP and immune, stromal, and ESTIMATE scores of the TME, indicating that TROAP plays different immune regulatory roles in various cancer types.

Furthermore, TME influences the therapeutic response and clinical outcome (51). TILs are an integral component of the TME and be correlated with prognosis and response to therapy in clinical practice, most notably in immune checkpoint inhibitor therapy (52). Particularly, TROAP expression correlated with all examined immune cell marker genes and immune-related genes, demonstrating the potential immune function of TROAP in THCA. Moreover, Correlation analysis showed a positive correlation between immune checkpoint genes and TROAP expression in the majority of tumor types, manifesting that TROAP may be involved in immune escape. Through immunosuppressive effects, Treg cells cause the body to develop antigenic tolerance to tumor cells, which will cause the immune escape of tumor cells. Therefore, Treg cells are also considered as a kind of immune cells that help tumors survive and promote their growth. Our analysis found a positive correlation between TROAP and Treg cells in 13 cancers. Further single-cell analysis of tumor immunity using the TISCH database uncovered a significant positive correlation between TROAP and Proliferating T cells in 26 tumors. Demonstrating that TROAP expression is closely associated with immune infiltration of tumor cells, affecting patient prognosis, and providing new targets for the development of immunosuppressive agents. Therefore, in the future, the expression level of TROAP can be used to assess the effect of immunotherapy. Moreover, based on traditional immunotherapy, targeted therapy against TROAP can be developed to improve efficacy.

Chemotherapeutic drugs are the primary treatment of choice for many cancers. Our analysis of drug databases revealed that TROAP expression is related to multiple anti-tumor drug sensitivity. These drugs constitute the therapeutic schedule for patients with different types of cancers. We will further explore the specific mechanisms by which TROAP is involved in chemotherapy resistance in different cancers, which will be important in guiding personalized clinical dosing decisions.

## Conclusion

Collectively, our comprehensive pan-cancer analysis showed the characteristics of TROAP in multiple ways, including expression pattern, survival prognosis, genetic mutation, TMB, MSI, MATH, immune TME, and drug resistance. As TROAP is abnormally expressed across multiple cancers and predicts worse outcomes in cancer patients, it might be an effective cancer therapy target. Our experiments manifested that TROAP knockdown inhibited cell proliferation and migration compared with the control group in HCC and CRC cells. Mechanism analysis revealed that TROAP silence could significantly downregulate the level of downstream target genes of PI3K/Akt/GSK-3β signaling, confirming that TROAP-induced malignant biological behavior and tumor progression *via* PI3K/Akt/GSK-3 signaling pathway. There was frequent amplification of the TROAP genomic region, which was correlated with TROAP expression. In addition, aberrant TROAP expression was associated with ESTIMATE score, MSI, TMB, and tumor immune microenvironment in cancers. Based on the results of this study, TROAP can be used as a new drug-resistance gene to predict the sensitivity of patients to chemotherapy drugs. In conclusion, the potential application of TROAP as a biomarker for predicting prognosis and response to immunotherapy. Therefore, for tumor patients with high TROAP expression, blocking TROAP might be a promising therapeutic modality.

## Data availability statement

The original contributions presented in the study are included in the article/**Supplementary Material**. Further inquiries can be directed to the corresponding author.

## Ethics statement

The studies involving human participants were reviewed and approved by the ethics committee of the Xiangya Hospital, Central South University, China. The patients/participants provided their written informed consent to participate in this study.

## Author contributions

ZL and FP had the idea and wrote the article. ZY and YZ performed the literature search and data analysis. ZP, YD, and NL drafted and critically revised the work. All authors contributed to the article and approved the submitted version.

## Funding

This work was supported by the National Natural Science Foundation of China (81873574), Natural Science Foundation of Hunan Province, China (2020JJ4905).

## Acknowledgments

We thank the staff members of NHC Key Laboratory of Cancer Proteomics for providing support and assistance in data collection and analysis.

## Conflict of interest

The authors declare that the research was conducted in the absence of any commercial or financial relationships that could be construed as a potential conflict of interest.

## Publisher’s note

All claims expressed in this article are solely those of the authors and do not necessarily represent those of their affiliated organizations, or those of the publisher, the editors and the reviewers. Any product that may be evaluated in this article, or claim that may be made by its manufacturer, is not guaranteed or endorsed by the publisher.

## Supplementary material

Supplementary Figure 1 The transcriptional expression of TROAP in different cancer cell lines and immune cells analyzed by the BioGPS database.

Supplementary Figure 2 The transcriptional expression of TROAP stratified by stage in **(A)** adrenocortical carcinoma(ACC), **(B)** bladder urothelial carcinoma(BLCA), **(C)** cholangio carcinoma(CHOL), **(D)** esophageal carcinoma(ESCA), **(E)** kidney chromophobe(KICH), **(F)** lung squamous cell carcinoma (LUSC), **(G)** rectum adenocarcinoma(READ), **(H)** stomach adenocarcinoma(STAD), **(I)** thyroid carcinoma(THCA), **(J)** uterine corpus endometrial carcinoma(UCEC), **(K)**l ung adenocarcinoma (LUAD), **(L)** kidney renal papillary cell carcinoma (KIRP), and **(M)** kidney renal clear cell carcinoma(KIRC).

Supplementary Figure 3 The difference in immunohistochemistry staining of TROAP between tumor tissues and the adjacent normal samples in pan-cancer. TROAP protein expression was significantly higher in **(A)** STAD, **(B)** UCEC, **(C)** colon adenocarcinoma (COAD), **(D)** LUAD, **(E)** LUSC, **(F)** liver hepatocellular carcinoma (LIHC), **(G)** BLCA, **(H)** breast invasive carcinoma (BRCA), **(I)** skin cancer, **(J)** ovarian serous cystadenocarcinoma (OV), **(K)** pancreatic adenocarcinoma (PAAD), and **(L)** head and neck cancer tissues than normal tissues, respectively.

Supplementary Figure 4 The association of TROAP transcriptional expression and survival possibility in pan-cancer determined by univariate Cox regression analysis. **(A)** overall survival, **(B)** disease-free survival, **(C)** disease-specific survival, and **(D)** progression-free interval.

Supplementary Figure 5 Kaplan-Meier **(K–M)** analysis of survival difference in high- and low- TROAP expression group in pan-cancer. **(A–K)** overall survival. **(L–W)** disease-free survival.

Supplementary Figure 6 The relationship between TROAP gene transcriptional expression and the prognosis of patients with different cancers from the GEO database analyzed by the PrognoScan database. Distant metastasis-free survival in breast cancer (**A**, GSE11121 dataset). Distant recurrence-free survival in soft tissue cancer (**B**, GSE30929 dataset). Relapse-free survival in breast cancer (**C**, GSE1456-GPL96 dataset) and lung cancer (**D**, GSE31210 dataset). Overall survival in skin cancer (**E**, GSE19234 dataset), breast cancer (**G**, GSE1456-GPL96 dataset), brain cancer (**I**, GSE1456-GPL96 dataset), bladder cancer (**K**, GSE13507 dataset), and lung cancer (**L**, GSE31210 dataset). Disease-specific survival in bladder cancer (**F**, GSE13507 dataset), breast cancer (**H**, GSE1456-GPL96 dataset), and blood cancer (**M**, GSE2658 dataset). Disease-free survival in breast cancer (**J**, GSE7378 dataset), and colorectal cancer (**N**, GSE14333 dataset).

Supplementary Figure 7
**(A–L)** The correlation of TROAP transcriptional expression and immune subtypes in **(A)** ESCA, **(B)** prostate adenocarcinoma(PRAD), **(C)** UCEC, **(D)** cervical squamous cell carcinoma and endocervical adenocarcinoma (CESC), **(E)** stomach adenocarcinoma(STAD), **(F)** OV, **(G)** head and neck squamous cell carcinoma(HNSC), **(H)**KIRC, **(I)** KIRP, **(J)** brain lower grade glioma (LGG),**(K)** PAAD, and **(L)** THCA using TISIDB database. **(M–T)** The relationship between TROAP transcriptional expression and molecular subtypes in **(M)** PRAD, **(N)** STAD, **(O)** UCEC, **(P)** glioblastoma multiforme (GBM), **(Q)** HNSC, **(R)** KIRP, **(S)** LGG, and **(T)** ACC using UALCAN online tool. C1 (wound healing); C2 (IFN-gamma dominant); C3 (inflammatory); C4 (lymphocyte depleted); C5 (immunologically quiet); C6 (TGF-b dominant). All*: P < 0.05; **:P < 0.01; ***: P < 0.001; ****: P < 0.0001; and ns: no significance.

Supplementary Figure 8 The correlation of the TROAP expression with the infiltration of six immune cell types, namely B cell, CD4+ T cell, CD8+ T cell, neutrophil, macrophage, and dendritic cell.

Supplementary Figure 9 The relationship between the transcriptional expression of TROAP and drug sensitivity in CTRP **(A)** and GDSC **(B)** databases, respectively. GDSC, Genomics of Drug Sensitivity in Cancer; CTRP, The Cancer Therapeutics Response Portal.

Supplementary Figure 10 Tumor immune single-cell analysis was performed on TROAP in the 26 cancers using the TISCH database.

## Abbreviations

TME, tumor microenvironment; MSI, microsatellite instability; TMB, tumor mutation burden; NEO, Neoantigen; ICIs, immune checkpoint inhibitors; OS, overall survival; DFS, disease-free survival; HPA, the human protein atlas; IHC, immunohistochemistry; MATH, mutant-allele tumor heterogeneity; CNV, copy number variations; MHC, major histocompatibility complex; IHC, immunohistochemistry staining; DSS, disease-specific survival; m6A, N6-methyladenosine; m1A, N1-methyladenosine; m5C, 5-methylcytidine; DC, dendritic cell; TILs, tumor-infiltrating lymphocytes; ICs, immune checkpoints.
