# Supplementary figures and images for: Pan-cancer analysis of trophinin-associated protein with potential implications in clinical significance, prognosis, and tumor microenvironment in human cancers
Source: Front Oncol. 2022 Nov 7;12:971618. doi: 10.3389/fonc.2022.971618 (PMC9677944; doi:10.3389/fonc.2022.971618)

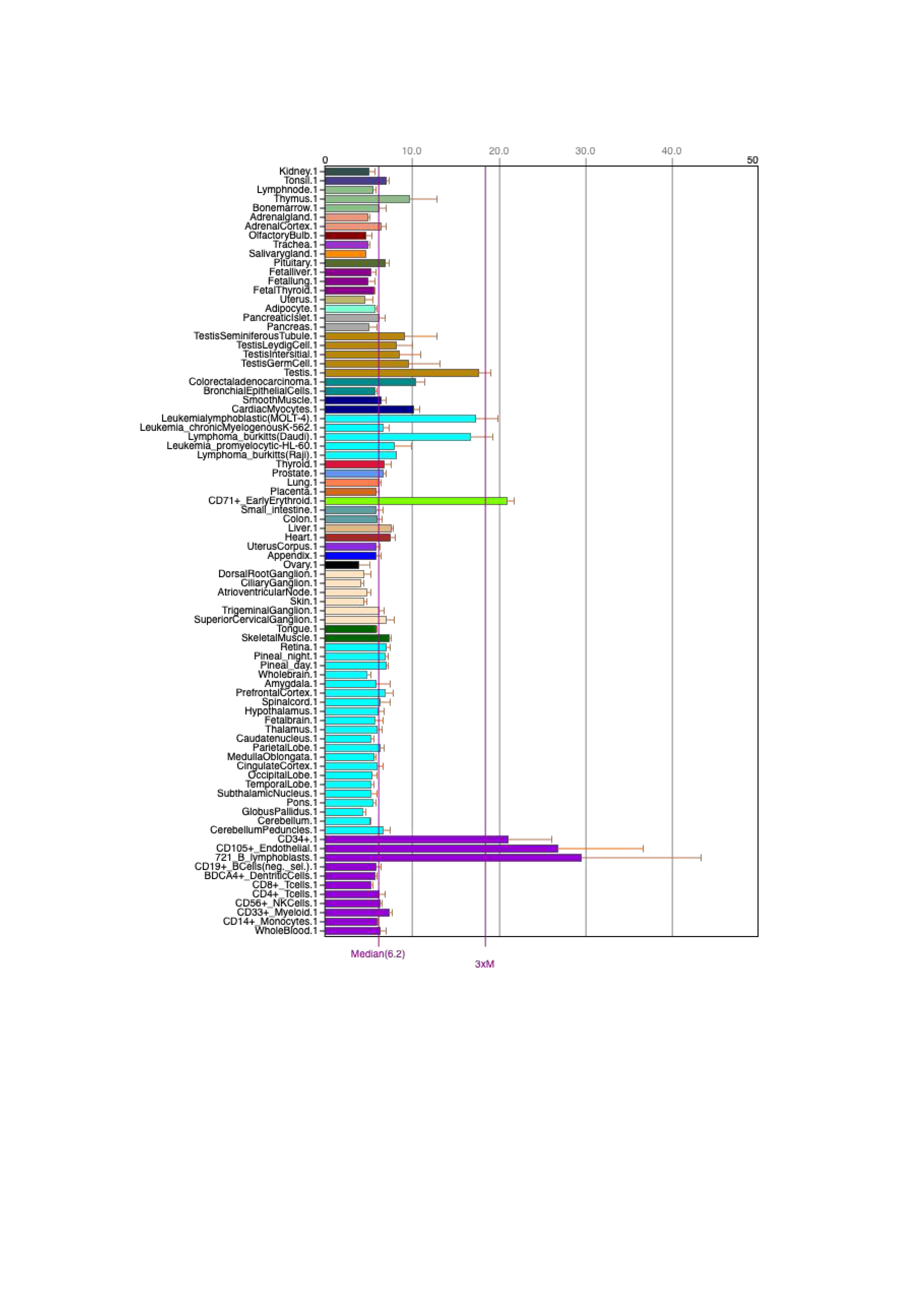

Supplement: Supplementary Figure 1 — The transcriptional expression of TROAP in different cancer cell lines and immune cells analyzed by the BioGPS database. [file Image_1.tif]

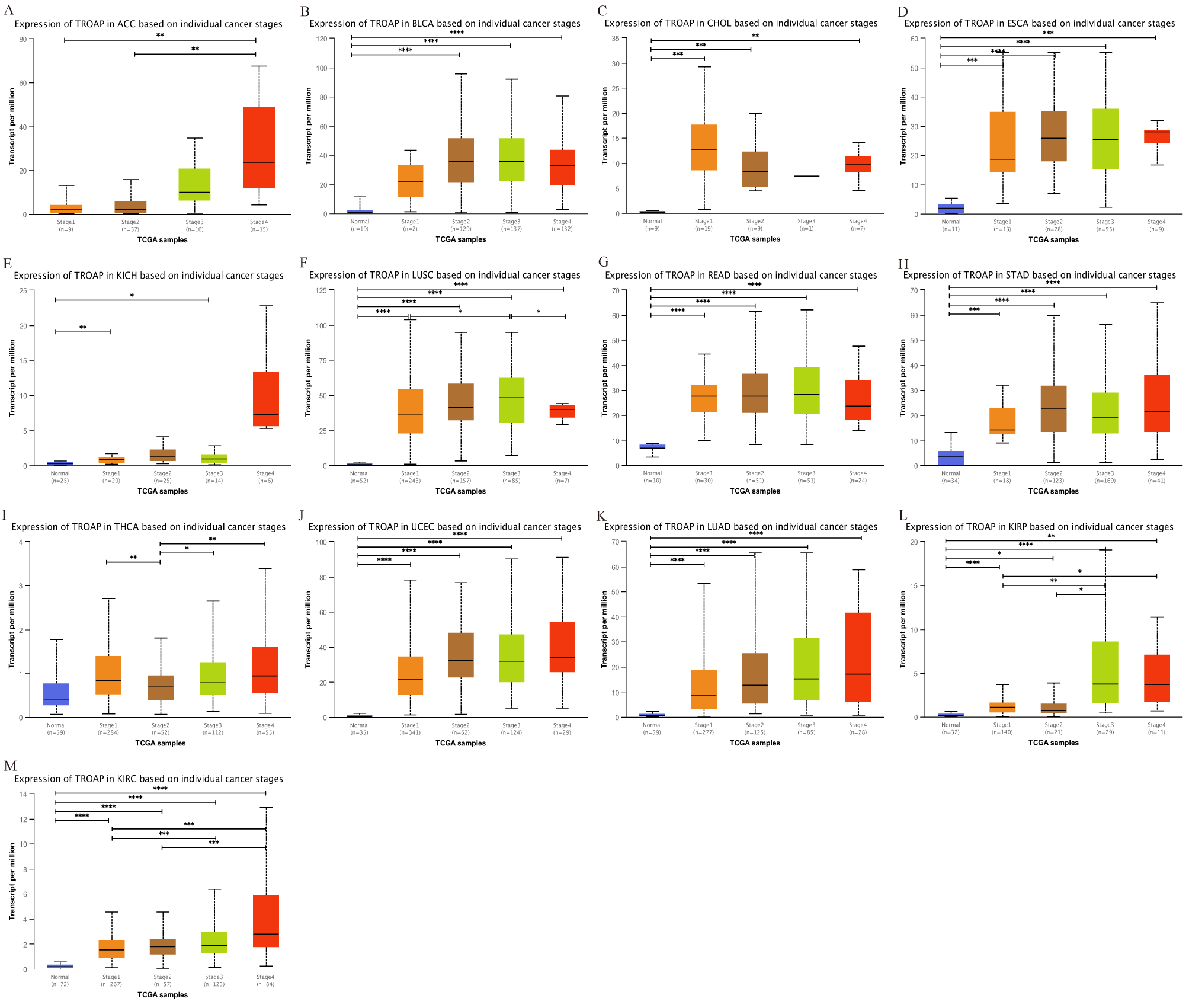

Supplement: Supplementary Figure 2 — The transcriptional expression of TROAP stratified by stage in (A) adrenocortical carcinoma(ACC), (B) bladder urothelial carcinoma(BLCA), (C) cholangio carcinoma(CHOL), (D) esophageal carcinoma(ESCA), (E) kidney chromophobe(KICH), (F) lung squamous cell carcinoma (LUSC), (G) rectum adenocarcinoma(READ), (H) stomach adenocarcinoma(STAD), (I) thyroid carcinoma(THCA), (J) uterine corpus endometrial carcinoma(UCEC), (K)l ung adenocarcinoma (LUAD), (L) kidney renal papillary cell carcinoma (KIRP), and (M) kidney renal clear cell carcinoma(KIRC). [file Image_2.tif]

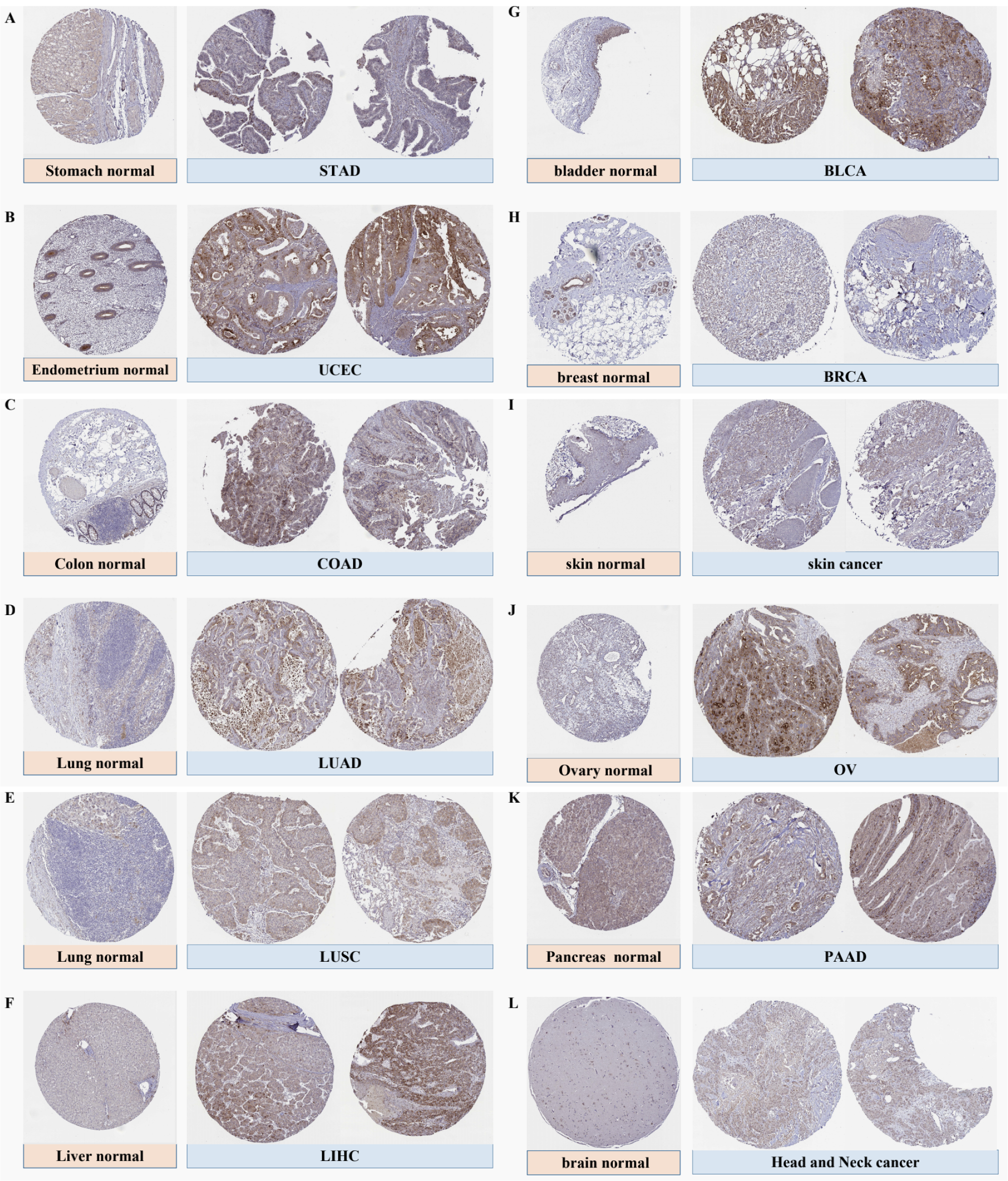

Supplement: Supplementary Figure 3 — The difference in immunohistochemistry staining of TROAP between tumor tissues and the adjacent normal samples in pan-cancer. TROAP protein expression was significantly higher in (A) STAD, (B) UCEC, (C) colon adenocarcinoma (COAD), (D) LUAD, (E) LUSC, (F) liver hepatocellular carcinoma (LIHC), (G) BLCA, (H) breast invasive carcinoma (BRCA), (I) skin cancer, (J) ovarian serous cystadenocarcinoma (OV), (K) pancreatic adenocarcinoma (PAAD), and (L) head and neck cancer tissues than normal tissues, respectively. [file Image_3.tif]

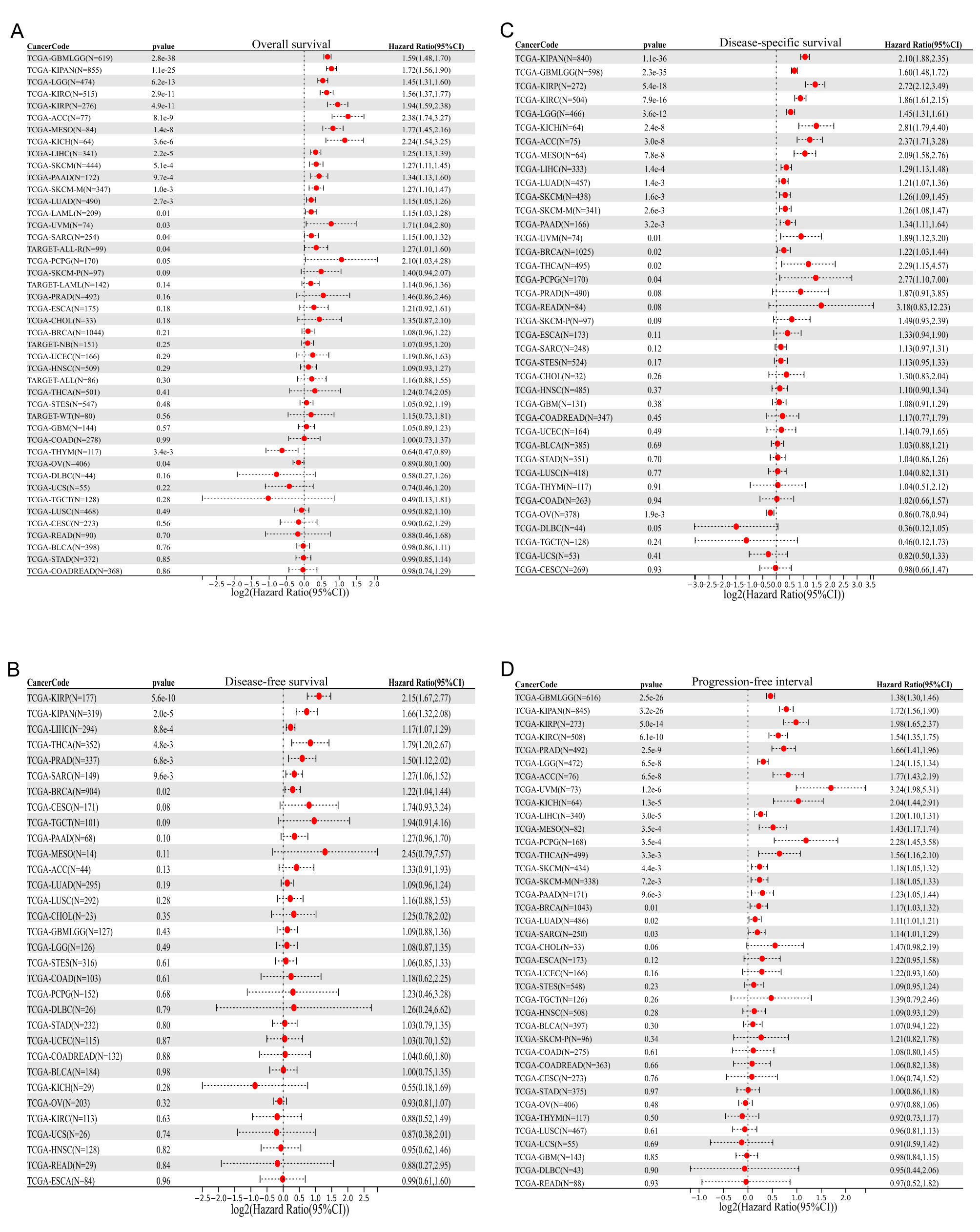

Supplement: Supplementary Figure 4 — The association of TROAP transcriptional expression and survival possibility in pan-cancer determined by univariate Cox regression analysis. (A) overall survival, (B) disease-free survival, (C) disease-specific survival, and (D) progression-free interval. [file Image_4.tif]

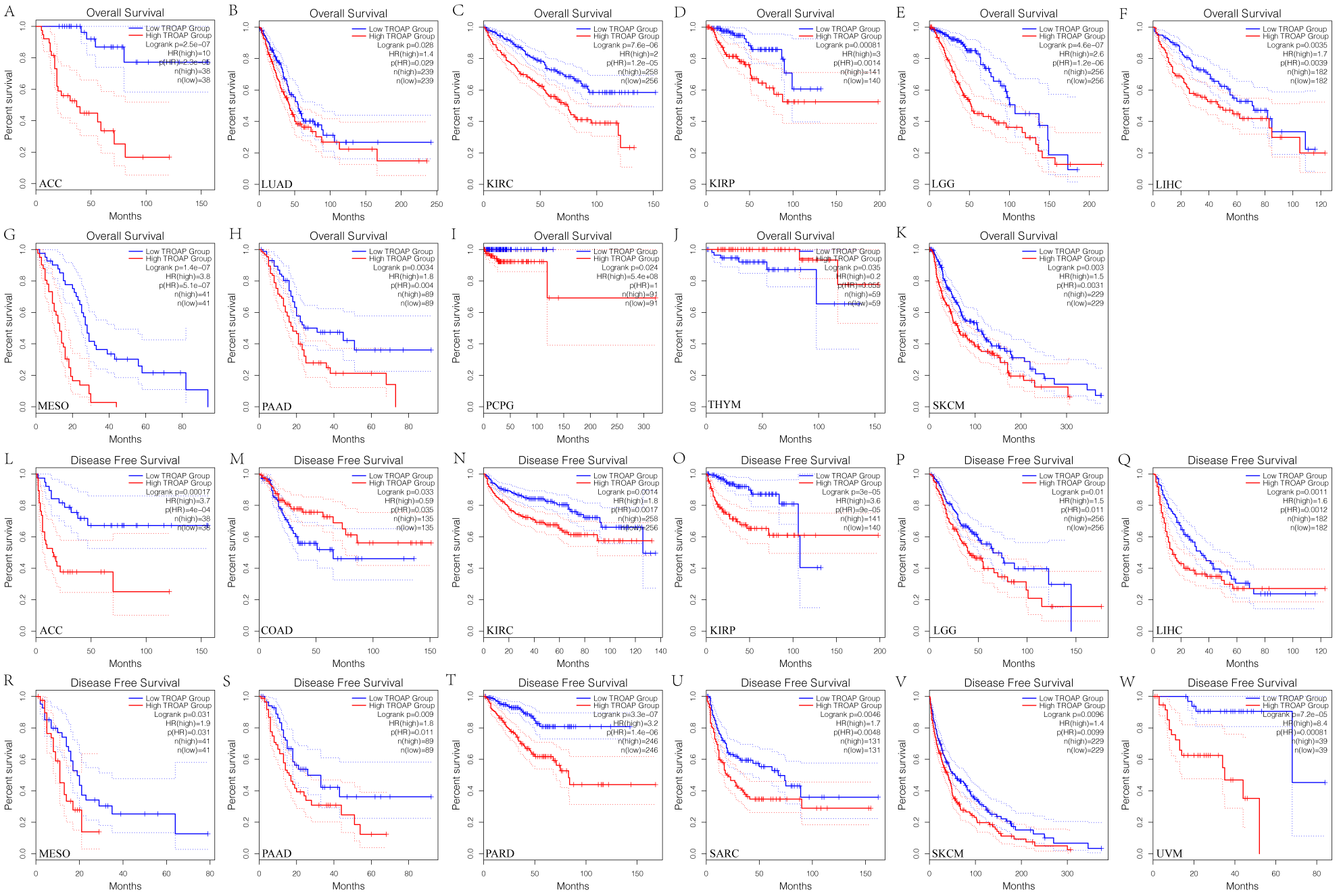

Supplement: Supplementary Figure 5 — Kaplan-Meier (K–M) analysis of survival difference in high- and low- TROAP expression group in pan-cancer. (A–K) overall survival. (L–W) disease-free survival. [file Image_5.tif]

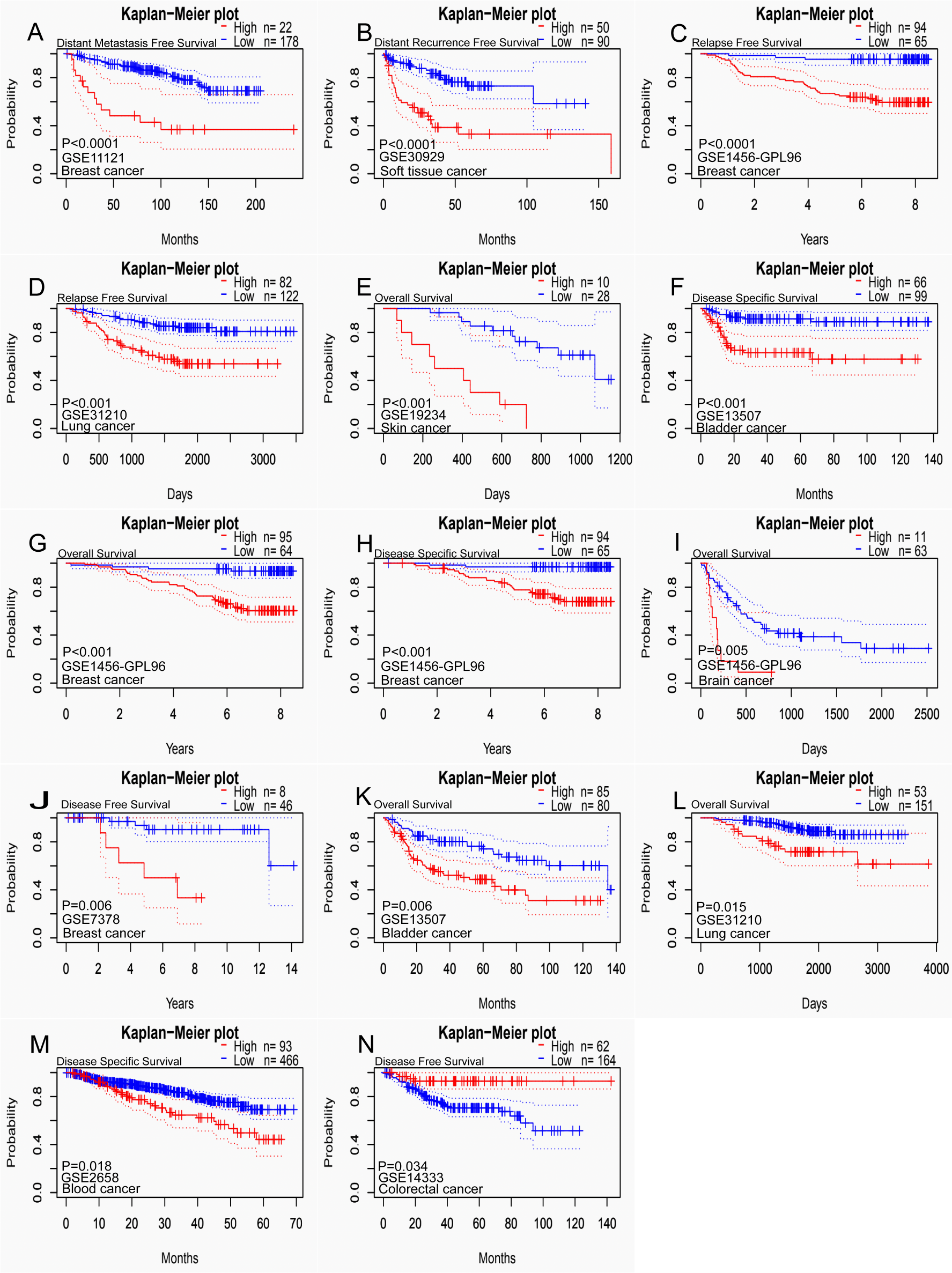

Supplement: Supplementary Figure 6 — The relationship between TROAP gene transcriptional expression and the prognosis of patients with different cancers from the GEO database analyzed by the PrognoScan database. Distant metastasis-free survival in breast cancer (A, GSE11121 dataset). Distant recurrence-free survival in soft tissue cancer (B, GSE30929 dataset). Relapse-free survival in breast cancer (C, GSE1456-GPL96 dataset) and lung cancer (D, GSE31210 dataset). Overall survival in skin cancer (E, GSE19234 dataset), breast cancer (G, GSE1456-GPL96 dataset), brain cancer (I, GSE1456-GPL96 dataset), bladder cancer (K, GSE13507 dataset), and lung cancer (L, GSE31210 dataset). Disease-specific survival in bladder cancer (F, GSE13507 dataset), breast cancer (H, GSE1456-GPL96 dataset), and blood cancer (M, GSE2658 dataset). Disease-free survival in breast cancer (J, GSE7378 dataset), and colorectal cancer (N, GSE14333 dataset). [file Image_6.tif]

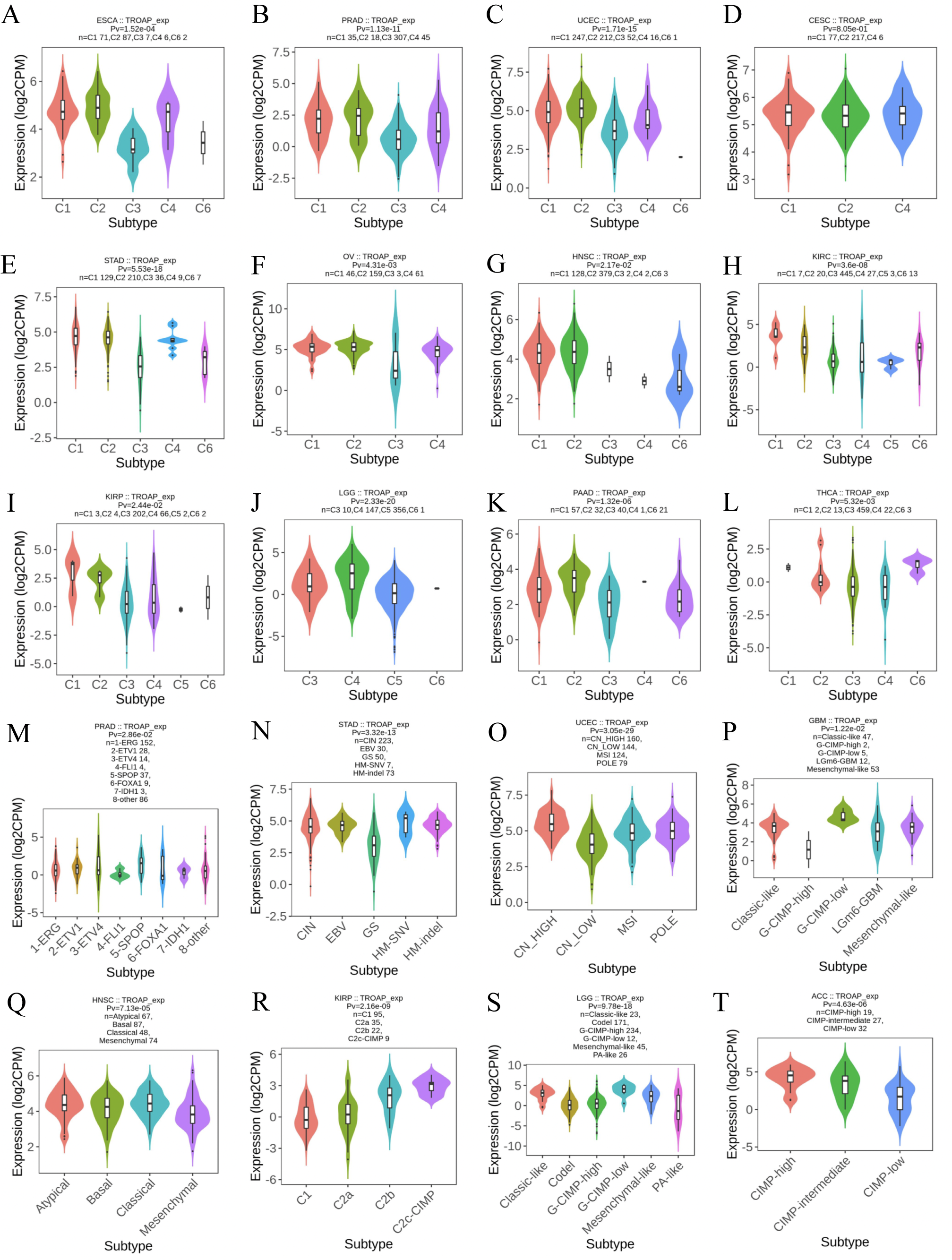

Supplement: Supplementary Figure 7 — (A–L) The correlation of TROAP transcriptional expression and immune subtypes in (A) ESCA, (B) prostate adenocarcinoma(PRAD), (C) UCEC, (D) cervical squamous cell carcinoma and endocervical adenocarcinoma (CESC), (E) stomach adenocarcinoma(STAD), (F) OV, (G) head and neck squamous cell carcinoma(HNSC), (H)KIRC, (I) KIRP, (J) brain lower grade glioma (LGG),(K) PAAD, and (L) THCA using TISIDB database. (M–T) The relationship between TROAP transcriptional expression and molecular subtypes in (M) PRAD, (N) STAD, (O) UCEC, (P) glioblastoma multiforme (GBM), (Q) HNSC, (R) KIRP, (S) LGG, and (T) ACC using UALCAN online tool. C1 (wound healing); C2 (IFN-gamma dominant); C3 (inflammatory); C4 (lymphocyte depleted); C5 (immunologically quiet); C6 (TGF-b dominant). All*: P < 0.05; **:P < 0.01; ***: P < 0.001; ****: P < 0.0001; and ns: no significance. [file Image_7.tif]

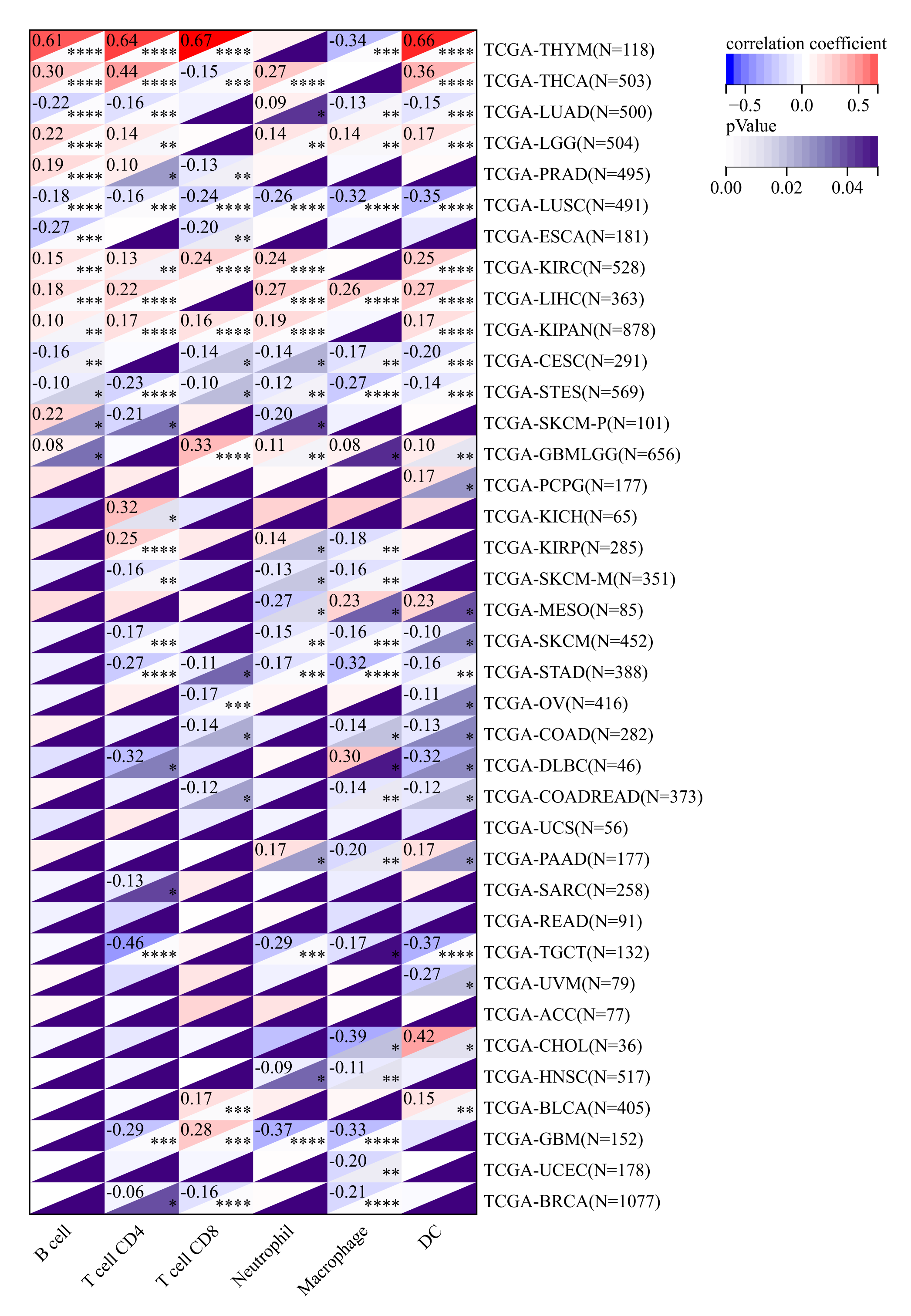

Supplement: Supplementary Figure 8 — The correlation of the TROAP expression with the infiltration of six immune cell types, namely B cell, CD4+ T cell, CD8+ T cell, neutrophil, macrophage, and dendritic cell. [file Image_8.tif]

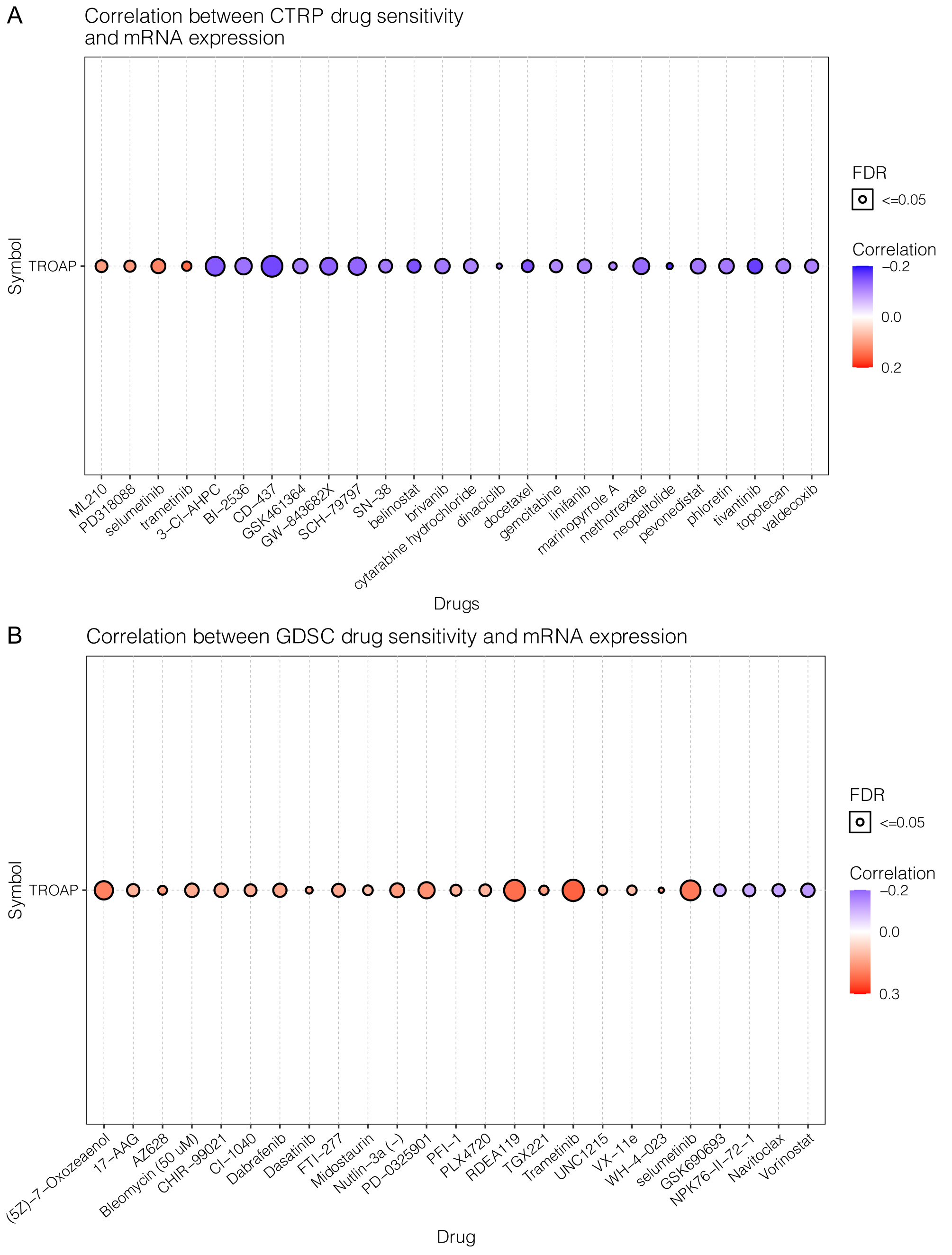

Supplement: Supplementary Figure 9 — The relationship between the transcriptional expression of TROAP and drug sensitivity in CTRP (A) and GDSC (B) databases, respectively. GDSC, Genomics of Drug Sensitivity in Cancer; CTRP, The Cancer Therapeutics Response Portal. [file Image_9.tif]

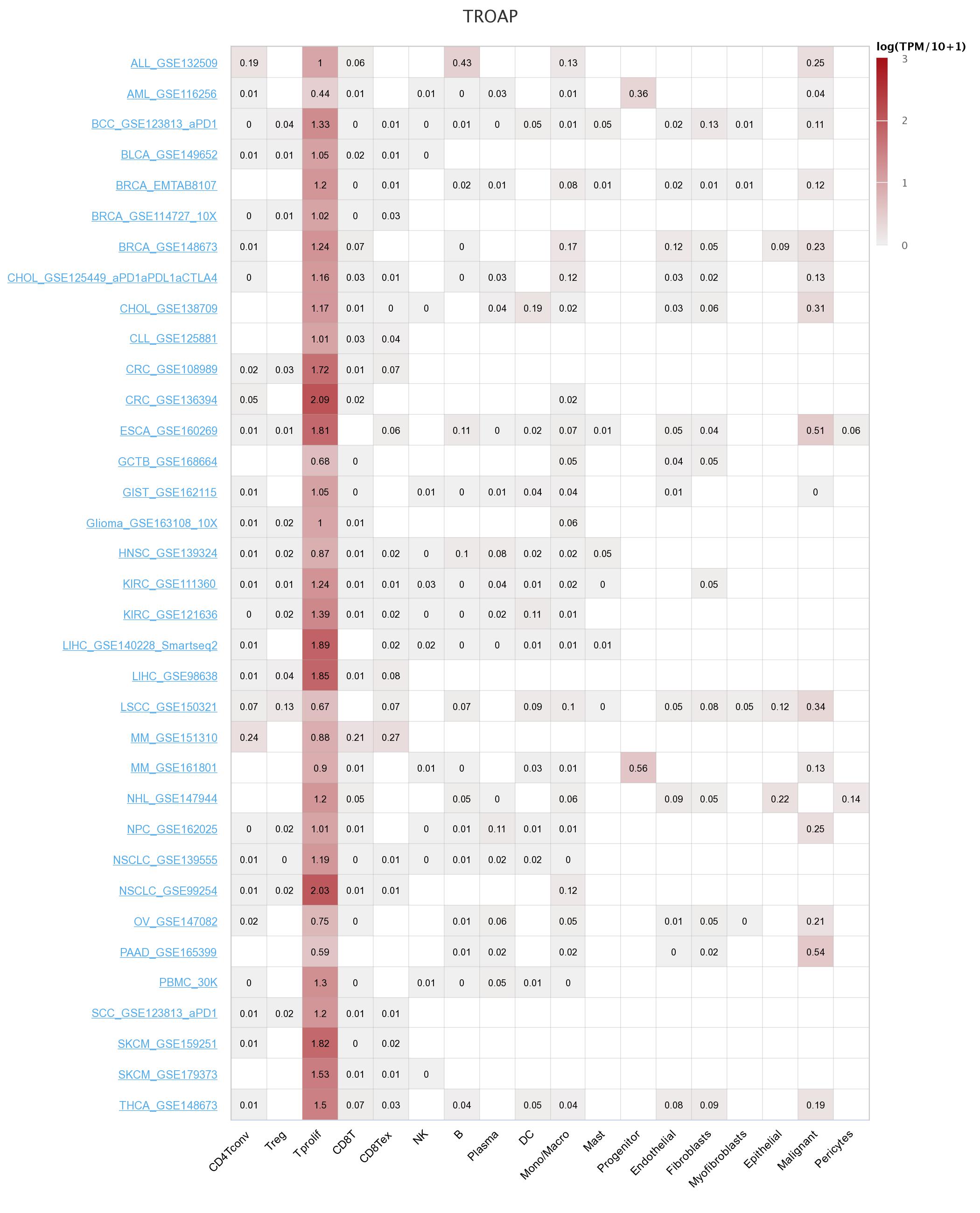

Supplement: Supplementary Figure 10 — Tumor immune single-cell analysis was performed on TROAP in the 26 cancers using the TISCH database. [file Image_10.jpeg]
